# Inshore and offshore marine migration pathways of Atlantic salmon post‐smolts from multiple rivers in Scotland, England, Northern Ireland, and Ireland

**DOI:** 10.1111/jfb.15760

**Published:** 2024-04-28

**Authors:** Jessica R. Rodger, Jessie Lilly, Hannele M. Honkanen, Diego del Villar, Richard Kennedy, Niall Ó. Maoiléidigh, Patrick Boylan, Robert Rosell, David J. Morris, Ross O'Neill, Catherine Waters, Deirdre Cotter, Lorna Wilkie, Andrea Barkley, Amy Green, Samantha V. Beck, Jamie Ribbens, Jim Henderson, Debbie Parke, Alan Kettle‐White, Lucy Ballantyne, Shona Marshall, Paul Hopper, Niall Gauld, Jason D. Godfrey, Lauren E. Chapman, James Thorburn, Alan Drumm, Fred Whoriskey, Brian Shields, Philip Ramsden, James Barry, Michael Millane, William Roche, John D. Armstrong, Alan Wells, Silas Walton, Melanie Fletcher, David M. Bailey, Bill Whyte, Ross McGill, Mark Bilsby, Ken Whelan, Colin W. Bean, Colin E. Adams

**Affiliations:** ^1^ Scottish Centre for Ecology and the Natural Environment, School of Biodiversity, One Health and Veterinary Medicine University of Glasgow Glasgow UK; ^2^ Atlantic Salmon Trust Perth UK; ^3^ Loughs Agency Londonderry Northern Ireland; ^4^ Agri‐food and Biosciences Institute Hillsborough Northern Ireland; ^5^ Marine Institute Newport Ireland; ^6^ Chief Scientific Advisor's Office Department of Agriculture, Environment and Rural Affairs (DAERA), Jubilee House Ballykelly UK; ^7^ Freshwater Fisheries Laboratory Marine Scotland Science Pitlochry UK; ^8^ Galloway Fisheries Trust Station Industrial Estate Newton Stewart UK; ^9^ The Nith Catchment Fishery Trust and Nith District Salmon Fishery Board Dumfries UK; ^10^ Argyll Fisheries Trust Inveraray UK; ^11^ Lochaber Fisheries Trust Torlundy UK; ^12^ West Sutherland Fisheries Trust The Gardeners Cottage Scourie UK; ^13^ Outer Hebrides Fisheries Trust Marybank UK; ^14^ Scottish Oceans Institute St Andrews UK; ^15^ School of Biology Queens University Belfast Belfast UK; ^16^ Department of Biology Dalhousie University Halifax Nova Scotia Canada; ^17^ Environment Agency Penrith UK; ^18^ Inland Fisheries Ireland Dublin Ireland; ^19^ Fisheries Management Scotland Edinburgh UK; ^20^ Natural England Penrith UK; ^21^ School of Biodiversity, One Health and Veterinary Medicine University of Glasgow Glasgow UK; ^22^ NatureScot Clydebank UK; ^23^ Present address: Northwest Atlantic Fisheries Centre, 80E Whitehills Road St. John's Newfoundland Canada

**Keywords:** collaboration, marine management, migratory, navigation, *Salmo salar*, smolts

## Abstract

The migratory behavior of Atlantic salmon (*Salmo salar*) post‐smolts in coastal waters is poorly understood. In this collaborative study, 1914 smolts, from 25 rivers, in four countries were tagged with acoustic transmitters during a single seasonal migration. In total, 1105 post‐smolts entered the marine study areas and 438 (39.6%) were detected on a network of 414 marine acoustic receivers and an autonomous underwater vehicle. Migration pathways (defined as the shortest distance between two detections) of up to 575 km and over 100 days at sea were described for all 25 populations. Post‐smolts from different rivers, as well as individuals from the same river, used different pathways in coastal waters. Although difficult to generalize to all rivers, at least during the year of this study, no tagged post‐smolts from rivers draining into the Irish Sea were detected entering the areas of sea between the Hebrides and mainland Scotland, which is associated with a high density of finfish aquaculture. An important outcome of this study is that a high proportion of post‐smolts crossed through multiple legislative jurisdictions and boundaries during their migration. This study provides the basis for spatially explicit assessment of the impact risk of coastal pressures on salmon during their first migration to sea.

## 1 INTRODUCTION

Anadromous Atlantic salmon (*Salmo salar*) from populations in Europe make long‐distance migrations from fresh waters, through coastal zones, mostly to areas of high resource availability in the northeast Atlantic Ocean (Friedland, 1998; Gilbey et al., 2021; Holm et al., 2000; Utne et al., 2022). They begin their first migration from their natal rivers to sea during the smolt stage of their life cycle, but on reaching marine waters, they are generally termed post‐smolts. The pathways that post‐smolts use to reach their feeding grounds, their migration behavior, and the cues that they use for navigation are poorly understood (Dadswell et al., 2010; Ounsley et al., 2020).

Trawl netting studies have captured post‐smolts, along the continental shelf edge, to the west of the UK and Ireland, in early summer and toward the end of their first summer at sea in an area around the Vøring plateau west of Norway (Holm et al., 2000). This suggests that salmon populations from rivers that drain to the west of the UK and Ireland are using the shelf edge as a migration route to feeding grounds in the Norwegian Sea (Gilbey et al., 2021). The migration pathways used by post‐smolts through coastal zones and onward to the shelf edge from these rivers are unclear. Several studies have linked post‐smolt migration pathways and the prevailing currents, suggesting that current‐following behavior may be important during migration (Dadswell et al., 2010; McIlvenny et al., 2021; Mork et al., 2012). However, there is also evidence that, at least, at times post‐smolt migration must involve active swimming. A particle tracking study by Ounsley et al. (2020) found that current‐following behavior alone did not explain the trajectory of migration of post‐smolts migrating from rivers in Scotland to reach their feeding grounds. Moriarty et al. (2016) suggested that directed swimming led to the highest migration success rate for Atlantic salmon through the Gulf of Maine. Similarly, in a study combining acoustic telemetry with particle tracking, Newton et al. (2021) showed that the actual migration route of post‐smolts in the coastal zone was best predicted by active swimming rather than by simply following the current. Therefore, it is highly likely that active navigation and swimming are required by most salmon post‐smolts during their early marine migration in the coastal zone. When post‐smolts reach better‐defined and more consistent oceanic currents, then a switch to current following may become the main form of navigation and orientation during migration (Jensen et al., 2022). Other factors are also likely to influence the migration pathways used. For example, the distribution of post‐smolts detected at sea may be linked with the presence and abundance of suitable prey items, suggesting that prey availability may influence migration pathways (Gilbey et al., 2021; Jensen et al., 2022; Utne et al., 2022).

Once salmon reach their presumed feeding area in the Norwegian Sea, studies have shown that fish from different populations aggregate (Gilbey et al., 2021; Hansen & Jacobsen, 2003). It is not known how early such an aggregation may develop and where different populations coalesce as they depart the coastal zone. The environmental conditions and sea currents that post‐smolts encounter when they first enter the marine environment vary considerably. Therefore, it seems likely that migration pathways would vary among populations, and potentially among individuals, reflecting spatial as well as temporal variation in the environmental conditions to which any individual post‐smolt may experience on entering coastal waters.

Much of the information available to date on post‐smolt marine migration patterns comes from mark‐recapture studies at sea, as well as, trawling studies that have used genetic markers or coded wire tags to assign post‐smolts back to their natal rivers (Gilbey et al., 2021; Harvey et al., 2019; Mork et al., 2012). These studies provide broad spatial distribution patterns of salmon post‐smolts at sea but provide relatively inexact positions for each individual fish, as the precise capture point along a trawl line is unknown. Trawl studies also do not provide definitive information on the migration pathways or the speed of the migration before capture. Telemetry has the capacity to provide spatially and temporally detailed information on the migration of individual salmon. A limitation of such studies is that they usually depend on strategically placed arrays of stationary receivers the number of which, their cost, and the complexity of their deployment logistics increase significantly with the distance from shore. As a result, until now, such studies have been largely conducted in estuarine and near‐coastal environments (but see Kocik et al., 2009; Lacroix et al., 2004, 2005; Chaput et al., 2019). Another approach that has been used to posit migration pathways is through simulation models built around ocean current models. Modeling, using high‐resolution oceanographic data has the potential to provide broad geographic coverage and high‐resolution outputs. However, the nature and role of the environmental cues used by salmon to navigate pathways are poorly understood, and the results of modeling studies to date are somewhat contradictory (contrast: Mork et al., 2012; Moriarty et al., 2016; Ounsley et al., 2020, McIlvenny et al., 2021, and Newton et al., 2021).

As marine coastal areas are subject to high levels of human activity, an understanding of the broad routes that salmon use as they migrate through coastal areas (hereafter migration pathways) has clear management importance where there may be a need to mitigate impacts. Coastal zones are increasingly used for renewable energy development (including wind, tidal, and wave energy), trawling, and aquaculture (Declerck et al., 2023; Scottish Government, 2020). Each of these activities constitutes a potential hazard where they overlap with migrating salmon. For example, potential impacts could include infestation with sea lice from aquaculture, exposure to increased mortality due to predator aggregation around power generating devices, and direct capture by fisheries (Bøhn et al., 2020; Copping et al., 2021; Finstad et al., 2000; ICES, 2004, 2020, 2023; Johnsen et al., 2021; Wyman et al., 2018).

The aim of the study presented here was to characterize the broad geographic scale patterns of movement of Atlantic salmon post‐smolts as they migrate through the immediate nearshore and offshore coastal environment around the west coasts of Scotland, northern England, Northern Ireland, and Ireland. To do this, we describe the migration pathways of 1105 post‐smolts from 25 rivers that entered the marine study area. This was made possible by merging data from multiple telemetry projects involving a collaboration by 21 different research groups. The data presented here provide the broad geographic patterns that emerge from this dataset; a sister paper (Lilly et al., 2023) examines more detailed questions around navigation cues and drivers of migration success using a subset of these data. The combination of these studies provides an important and unique insight into the previously unknown migratory pathways of salmon post‐smolts.

## 2 METHODS

**FIGURE 1:**
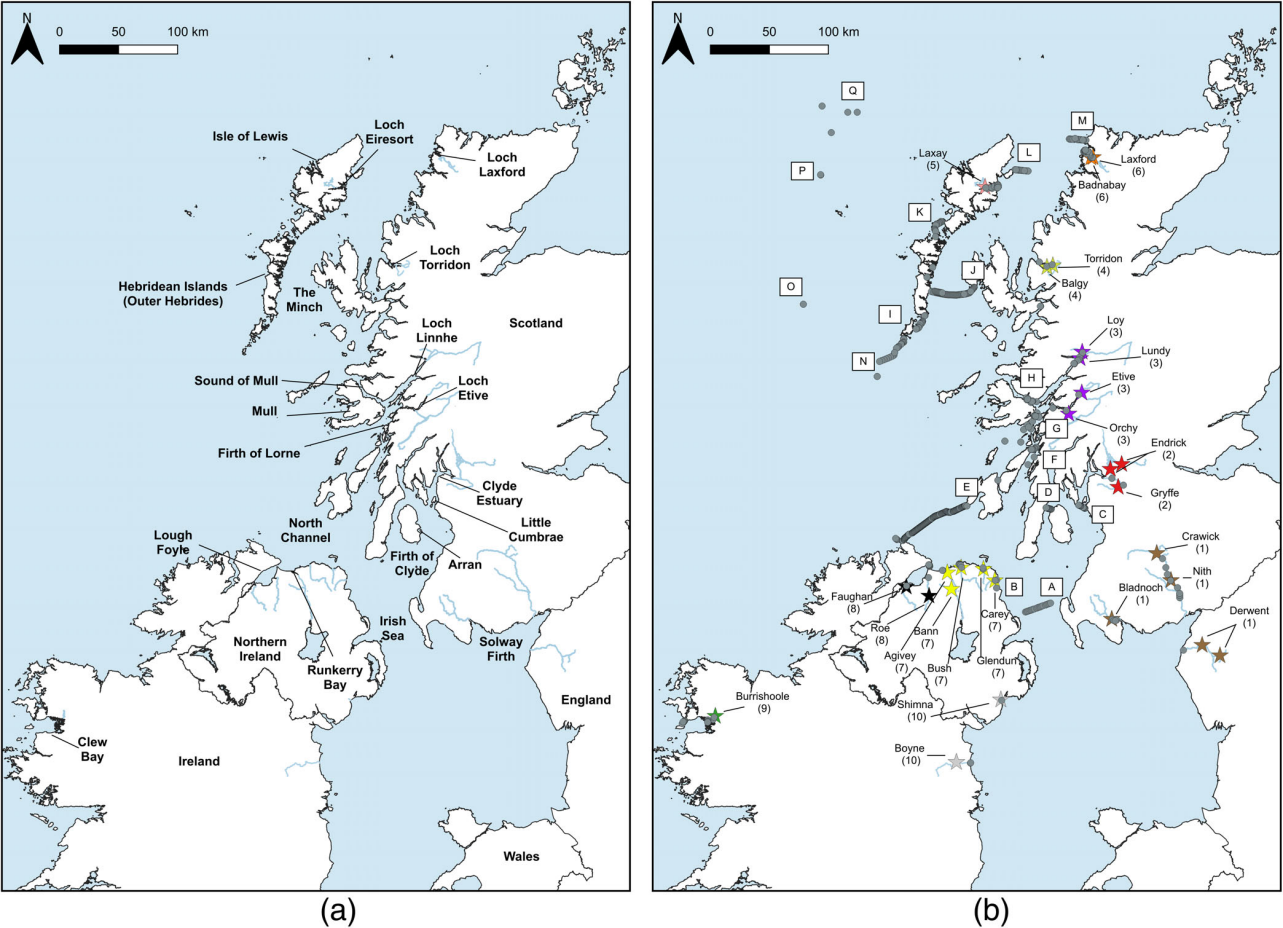
Maps of study area. (a) Map containing geographic names of areas included in this study; (b) map displaying the rivers (*N* = 25) where smolts (*N* = 1914) were tagged in Scotland, England, Northern Ireland, and Ireland for this study. Release sites are represented by stars, and acoustic receivers recovered (*N* = 370) are represented by gray dots. Marine monitoring lines and points (*N* = 17) for this project are labeled in alphabetical order from south to north (A–Q).

Eight acoustic telemetry projects focusing on Atlantic salmon and two additional projects using complimentary telemetry techniques (but not focusing on salmon directly) conducted in 2021 contributed data to the study presented here (see Table S1). All acoustic tags and fixed‐position acoustic receivers deployed operated on 69 kHz. Therefore, there was compatibility of tags and receiver detections between all projects. All studies were conducted in inshore and offshore marine waters (as defined in Marine Management Organisation, 2019) of western and eastern Ireland, north‐western England, north Northern Ireland, and western Scotland (including the area of sea to the west of the Outer Hebrides (Figure 1b). Combined, these projects covered a broad geographic area, spanning a latitudinal distance of ca. 480 km and a longitudinal distance of ca. 550 km (Figure 1b).

### 2.1 Fish capture and tagging

During the months of April and May 2021, 1854 wild Atlantic salmon smolts were captured across 25 rivers in the four jurisdictions comprising Scotland, England, Northern Ireland, and Ireland using 1.5‐m‐diameter rotary screw traps, fyke nets, Wolf‐type, downstream traps, and rod‐and‐line (River Shimna smolts only) (Figure 1b; Table 1). A further 60 hatchery‐reared Atlantic salmon smolts of a strain used in salmon ranching (see Cotter et al., 2022 for ranch stock information) were used at the River Burrishoole in Ireland. Therefore, 1914 Atlantic salmon smolts were tagged in this study. All smolts were tagged with acoustic tags and released into their natal rivers. There was a single release site in each river except for the rivers Derwent and Endrick, where two release sites were used (Table 1; Figure 1). Fish migrating from multiple tributaries or release sites in the same river system were considered together as fish from a single population. Therefore, smolts from the rivers Nith (mainstem) and Crawick (a Nith tributary) were combined and hereafter described as River Nith fish. Similarly, fish from the rivers Lundy and Loy were combined as the River Lochy group and from the rivers Agivey and Bann as the River Bann group. Also combined were data from fish from the multiple release sites on the rivers Endrick and Derwent.

**TABLE 1:** The river of origin, fish release site (latitude and longitude), type of tag used at that site, the tag life in days (provided by suppliers), nominal delay between acoustic transmissions, the number of fish entering the study (the total number tagged), the date of tagging, the tag expiry date range (which depends on tagging date and tag life), mean fish fork length (mm ± SD), mean weight (g ± SD), and median tag burden (determined as the weight in air of the tag relative to the weight of the fish) are provided only for those smolts that were successfully detected on the final riverine receiver and included in this study.

River	Release site (lat, long °)	Tag type	Tag life (days)	Nom. delay (s)	Fish number (Total tagged)	Date tagged	Tag expiry date	Median fork length (mm ± SD)	Median weight (g ± SD)	Median tag burden (± SD)
Scotland										
Endrick	56.0492, −4.4399 56.0085, −4.5897	V7‐2x	75	18–38	50 (145)	15‐04 to 03‐05	29‐06 to 17‐07	142 ± 8.8	29.1 ± 5.6	0.06 ± 0.01
Gryffe	55.8693, −4.4942	V7‐2x	75	18–38	93 (102)	12‐04 to 24‐04	26‐06 to 08‐07	14 ± 10.1	33.4 ± 6.7	0.05 ± 0.01
Bladnoch	54.8672, −4.4989	V7‐2x	75	18–38	53 (130)	20‐04 to 14‐05	04‐07 to 28‐07	141 ± 7.9	29.5 ± 5.1	0.06 ± 0.01
Nith	55.3783, −3.9313	V7‐2x	75	18–38	66 (130)	23‐04 to 06‐05	07‐07 to 20‐07	148 ± 10.9	32.8 ± 7.2	0.05 ± 0.01
Etive	56.5852, −5.0233	V7‐2x	75	18–38	62 (87)	19‐04 to 14‐05	03‐07 to 28‐07	137 ± 7.1	24.0 ± 3.7	0.07 ± 0.01
Orchy	56.4166, −5.1921	V7‐2x	75	18–38	88 (113)	16‐04 to 09‐05	05‐07 to 23‐07	137 ± 6.9	24.0 ± 3.8	0.07 ± 0.01
Lundy	56.8420, −5.0688	V7‐2x	75	18–38	36 (75)	12‐04 to 18‐05	26‐06 to 01‐08	135 ± 5.6	23.0 ± 3.5	0.07 ± 0.01
Loy	56.8907, −5.0347	V7‐2x	75	18–38	65 (170)	19‐04 to 10‐05	03‐07‐24‐07	134 ± 5.0	22.2 ± 2.8	0.07 ± 0.01
Badnabay	58.3722, −5.0450	V7‐2x	75	18–38	4 (9)	29‐04	13‐07	133 ± 11.6	25.3 ± 8.8	0.06 ± 0.01
Laxford	58.3760, −5.0101	V7‐2x	75	18–38	62 (91)	28‐04 to 05‐05	01‐07 to 19‐07	141 ± 8.9	26.6 ± 5.9	0.06 ± 0.01
Laxay	58.1041, −6.5480	V7‐2x	75	18–38	64 (119)	19‐04 to 05‐05	03‐07 to 19‐07	146 ± 13.5	31.8 ± 8.8	0.05 ± 0.01
Crawick	55.3783, −3.9313	V7‐2x	75	18–38	24 (50)	16‐04 to 23‐04	30‐06 to 07‐07	139 ± 5.1	27.2 ± 2.3	0.06 ± 0.01
Torridon	57.5397, −5.5146	ID‐LP6	70	20–30	6 (8)	20‐04 to 12‐05	29‐06 to 21‐07	135 ± 4.1	24.7 ± 1.9	0.05 ± 0.01
		ID‐LP7	101	20–30	2 (3)	30‐04	09‐08	139 ± 3.5	26.5 ± 1.7	0.07 ± 0.004
Balgy	57.5308, −5.5962	ID‐LP6	70	20–30	10 (12)	28‐04 to 21‐05	09‐07 to 30‐07	149 ± 11.9	31.6 ± 7.3	0.05 ± 0.01
		ID‐LP7	101	20–30	36 (51)	20‐04 to 18‐05	30‐07 to 27‐08	153 ± 14.7	35.8 ± 10.5	0.05 ± 0.01
England										
Derwent	54.6105, −3.0616, 54.6876, −3.2978	V7‐2x	75	18–38	41 (150)	29‐04 to 03‐05	13‐07 to 17‐07	140 ± 7.3	29.3 ± 5.0	0.05 ± 0.01
Northern Ireland										
Bush	55.2029, −6.5233	V7‐4L	522	30–60	73 (80)	13‐04 to 26‐04	17‐09 to 30‐09‐2022	168 ± 8.6	47.3 ± 8.0	0.04 ± 0.01
Glendun	55.1215, −6.0663	V7‐2x	99	20–40	21 (24)	16‐04 to 30‐04	07‐24 to 07‐08	142 ± 7.5	32.0 ± 4.6	0.05 ± 0.01
Bann	54.9841, −6.5618	V7‐2x	99	20–40	18 (59)	07‐05 to 25‐05	14‐08 to 01‐09	161 ± 15.5	43.0 ± 13.9	0.04 ± 0.01
Agivey	54.9879, −6.6661	V7‐2x	99	20–40	16 (41)	20‐04	28‐07	153 ± 9.3	37.0 ± 7.1	0.04 ± 0.01
Carey	55.2010, −6.2292	V7‐2x	99	20–40	7 (9)	29‐04 to 05‐05	06‐08 to 12‐08	163 ± 4.8	43.0 ± 3.5	0.03 ± 0.003
Roe	54.9710, −6.9253	V7‐2x	94	30–60	9 (11)	29‐04	01‐08	152 ± 3.9	36.0 ± 3.3	0.04 ± 0.004
Faughan	55.0251, −7.2359	V7‐2x	94	30–60	38 (53)	07‐05 to 15‐05	09‐08 to 17‐08	144 ± 4.2	‐	‐
Ireland										
Burrishoole	53.9137, −9.5713	V8‐4x	173	40–80	46 (50)	05‐05 to 07‐05	25‐10 to 27‐10	196 ± 9.6	85.4 ± 13.5	0.02 ± 0.004
		V7D‐2x	100	30–90	9 (10)	07‐05	15‐08	205 ± 17.8	109.7 ± 28.5	0.02 ± 0.01
		V7‐2x	120	40–80	19 (25)	05‐05 to 11‐05	04‐09 to 08‐09	148 ± 7.1	30.8 ± 4.0	0.05 ± 0.01
Boyne	53.721, −6.429	V7T–2x	75	15–45	6 (8)	15‐04 to 23‐04	14‐06 to 22‐06	156 ± 7.1	36.7 ± 3.7	0.04 ± 0.004
		V7TP‐4 L	102	20–60	38 (42)	16‐04 to 05‐05	27‐07 to 15‐08	162 ± 10.0	43.4 ± 9.6	0.05 ± 0.009
		V8‐4x	160	20–60	40 (50)	23‐04 to 07‐05	30‐09 to 14‐10	156 ± 10.3	36.8 ± 8.6	0.06 ± 0.01
Shimna	54.2112, −5.8914	V7‐2x	99	15–45	1 (3)	27‐04	04‐08	140 ± 8.7	31.3 ± 5.8	0.05 ± 0.008
		V7‐4 L	522	20–60	2 (4)	27‐04; 06‐05	01‐10 to 10‐10‐2022	150 ± 5.9	35.2 ± 3.4	0.05 ± 0.005

*Note*: For the River Burrishoole ranched smolts, which were on average larger than wild smolts, were tagged with V8‐4x and V7D‐2x tags, whereas wild smolts were tagged with V7‐2x tags. As weight was not recorded for smolts tagged on the River Faughan, tag burden could not be calculated. This table expands on the data presented in a sister paper (Lilly et al., 2023).

The majority of smolts were tagged with V7‐2x acoustic tags (see Table 2 for tag specifications; nominal delay range 18–60 s). Only smolts >130 mm fork length and 20 g weight were selected for tagging with such tags. This was to ensure that tag burden was kept < 8% of the fish body weight (Brown et al., 1999; Lennox et al., 2022; Newton et al., 2018). Smolts from the rivers Bush, Boyne, and Shimna were tagged with V7‐4L (nominal delay range 35–55 s) acoustic tags, and ranched smolts from the River Burrishoole and wild smolts from the rivers Boyne and Shimna that exceeded 175 mm fork length were tagged with V8‐4x, V7TP‐4 L, V7D‐2x, and V7T‐2x acoustic tags (Tables 1 and 2; nominal delay range 40–80, 20–60, 30–90, and 15–45 s, respectively). Wild salmon smolts from the rivers Balgy and Torridon were tagged using ID‐LP7 and ID‐LP6 (nominal delay range 20–30 s) acoustic tags. All tag types were transmitted on the 69‐kHz acoustic frequency and on code map 114 or 115 and, therefore, were compatible with all receivers deployed by each project involved in this study. The procedure for acoustic tagging followed standardized methods. In general, once anaesthetized (with MS222), smolts were measured for fork length (± 1 mm) and weight (± 0.1 g). An acoustic tag was then inserted into the abdominal cavity through a small incision anterior to the pelvic girdle. The incision was then closed with one or two interrupted surgeon knots using veterinary sutures. The tagged smolt was then placed in aerated water and released once fully recovered (see Lilly et al., 2021 for details). In the River Burrishoole, fish were held overnight in covered, flow‐through tanks before release the following day.

**TABLE 2:** The acoustic tag types used in this study, their supplier, and their specifications.

Tag type	Supplier	Tag diameter/length (mm)	Tag weight in air (g)	Power output (dB re 1μPa @ 1 m)	Transmission interval range (s)
V7‐2x	InnovaSea (Canada)	7/19.5	1.5	137	18–60
V7‐4L	InnovaSea (Canada)	7/21.5	1.8	137	35–55
V7D‐2x	InnovaSea (Canada)	7/21.5	1.8	137	30–90
V7T‐2x	InnovaSea (Canada)	7/19.5	1.5	137	15–45
V7TP‐4 L	InnovasSea (Canada)	7/23	1.9	137	20–60
V8‐4x	InnovaSea (Canada)	8/20.5	2.0	144	40–80
ID‐LP6	Thelma Biotel (Norway)	6/14.5	1.2	137	20–30
ID‐LP‐7	Thelma Biotel (Norway)	7.3/17	1.8	139	20–30

### 2.2 Ethical statement

The care and acoustic tagging of salmon smolts complied with animal welfare laws, guidelines, and polices. This work was conducted under license from national authorities in the UK and Ireland (UK Home Office license: PP0483054; PPL2869; 70/8928; PPL2913; PP3525229 & HPRA licenses: AE19121/P003; AE19118/P011).

### 2.3 Acoustic receiver deployment

In total, 414 acoustic receivers operating on 69 kHz (397 Innovasea, Canada [VR2W, VR2Tx, and VR2AR] models, 17 TRP 700 Thelma Biotel, Norway receivers) were deployed in this study; of these, 370 were subsequently recovered to provide useful data (only recovered receivers are shown in Figure 1b) (see also Tables 3 and S2 for more detailed information). Multiple acoustic receivers located adjacent to one another in a continuous detection line are henceforth termed a monitoring line; a single acoustic receiver is referred to as a monitoring point (Figure 1).

**TABLE 3:** A summary of receiver deployment (monitoring lines and monitoring points), location, including the midpoint latitude and longitude for each monitoring line or point, the number of receivers deployed and recovered, approximate distance (in kilometers) covered by monitoring line, and mean distance (in kilometers) between receivers in each monitoring line.

ID	Description of location	Latitude (°N)	Longitude (°W)	Number of receivers recovered (number of receivers deployed)	Approximate distance (km) covered by monitoring line	Mean distance (km) between receivers in monitoring line
A	Larne to Portpartrick	54.892	−5.640	20 (22)	23	1.0
B	Waterfoot, NI	55.064	−6.041	1 (1)	‐	‐
C	Little Cumbrae	55.725	−5.000	6 (8)	5.5	0.6
D	Isle of Arran	55.694	−5.437	6 (8)	6	0.65
E	Malin Head to Isle of Islay	55.494	−6.886	99 (108)	63	0.6
F	Isle of Jura to mainland Scotland	55.883	−6.108	7 (11)	7.5	0.7
G	Firth of Lorne	56.383	−5.620	10 (12)	5	0.7
H	Sound of Mull	56.512	−5.767	7 (8)	2.5	0.7
I	Southern Hebridean islands	56.989	−7.371	18 (26)	15	0.7
J	Isle of South Uist to mainland Scotland	57.269	−6.863	59 (71)	40	0.7
K	Northern Hebridean islands	57.741	−7.204	18 (18)	13	0.7
L	Isle of Lewis	58.249	−6.047	8 (12)	12	1
M	Sutherland	58.506	−5.248	17 (18)	14.5	1
N	North Atlantic Ocean (south of Hebridean islands)	56.604	−7.855	1 (1)	‐	‐
O	North Atlantic Ocean (west of Hebridean islands)	57.098	−8.969	1 (1)	‐	‐
P	North Atlantic (continental shelf)	58.0918	−8.913	1 (1)	‐	‐
Q	North Atlantic (continental shelf) autonomous underwater vehicle (AUV)	58.584	−8.614	1 (1)	‐	‐

In addition to stationary acoustic receivers deployed for this project, a submersible glider (autonomous underwater vehicle [AUV]) (Slocum G3 Glider, Teledyne Marine, USA) was deployed along the slope of the continental shelf to the west of the Outer Hebrides (Figure 1b, point Q). The glider was deployed from the *MRV Celtic Explorer* on April 16, 2021, at latitude 58.29693° N, longitude 9.11746° W and was subsequently retrieved on June 12, 2021. Pre‐programmed waypoints were selected to create a transect course based on suspected areas of post‐smolt congregation on the shelf edge, identified during the SALSEA MERGE research project (Utne et al., 2022). Pre‐programmed glider dive depth was restricted to 300 m when within the shelf edge area to ensure that it would remain within the detection range of post‐smolts moving in the surface waters. The submersible glider was fitted with a VMT acoustic receiver (Innovasea, Canada), operating on an acoustic frequency of 69 kHz, mounted externally, and, therefore, capable of detecting the acoustic tags used in this study. The submersible glider covered a transect with a total length of 1200 km over 57 days. The initial transect ran from west‐south‐west to east‐northeast along the shelf edge, with the glider then moving northwards off the shelf edge into deeper water. Strong off‐shelf currents meant that the glider could only re‐join the shelf after moving back west toward the start of the initial transect location. The glider then completed another transect before traveling into shallow coastal waters to the west of the Isle of Harris for recovery.

To detect the transition of smolts from the riverine to marine waters (sea lochs [fjords], coastal embayments, or estuaries), receivers were deployed close to where rivers discharged into marine waters (Table S2; Figure 1). Monitoring lines were also deployed at the exit of sea lochs (in Loch Etive, Loch Linnhe, Loch Eireasort, Loch Laxford, and Loch Torridon) and at the entrance and exit points of estuaries and coastal embayments (the Firth of Clyde, Lough Foyle, Runkerry Bay, and Clew Bay) (see Table S2 for further details), as well as in key locations in coastal waters (Figure 1b; Table 3). The detection distances covered by marine monitoring lines ranged from 5 to 63 km and the spacing between receivers from 0.6 to 1 km (Table 3). In the majority of cases, monitoring lines extended the full width of a channel; however, some monitoring lines (A, M, and L, Figure 1b) only partially covered the channel. The detectability of acoustic tags varies depending on the type of water (i.e., fresh water vs. saltwater) and local environment (e.g., noise reduces the detection range of tags; Reubens et al., 2019). Previous studies conducted in coastal marine waters, similar to those in this study, demonstrated detection ranges for V7 tags of 190–400 m (Main, 2021; Newton et al., 2021). Around 10% of receivers deployed were lost in this study. Therefore, an unknown proportion of tagged fish may have passed through monitoring line(s) undetected.

### 2.4 Data handling approach

For river systems discharging into estuaries and coastal embayments, only tags that were detected on a receiver at the mouth of each river, or on a receiver in the coastal marine environment, were included in further analysis. For river systems discharging into sea lochs, only tags that were detected at, or beyond, the monitoring line at the exit of the sea loch were included in the further analysis (Table 1). To remove possible false detections resulting from tag collisions or environmental noise, the raw data were filtered using the *false_detections* function in the R package *Glatos* (Holbrook et al., 2018). Therefore, fish detections were retained only if they were detected more than once on a single receiver, and the time delay between detections was between the minimal nominal delay and 30× the maximum nominal delay of the tag. In addition, tag detections that showed evidence of unrealistic post‐smolt behavior, such as swim speed or long residency events at a receiver that could have resulted from tag loss or the detection of a tag in a predator, were removed from further analysis. In total, two fish were deemed to have been predated (one from the Bann and one from the Boyne) and were removed from the analysis. An assumption of this study is that those remaining detections were of tags in migrating Atlantic salmon and are hereafter referred to as post‐smolts. Summary statistics including mean fork length (mm), mean weight (g), and mean tag burden were calculated for smolts from each river (Table 1). Tag burden was defined as the ratio between the weight of the acoustic tag in air (g) and fish weight (g).

### 2.5 Migration pathways

To determine the broader pattern of post‐smolt passage through inshore and offshore marine waters, migration pathways for individual fish were determined. Here we define a migration pathway as the minimum possible marine distance traveled by a post‐smolt between successive detection points. Therefore, a pathway can be determined only when a post‐smolt is detected at two or more monitoring points or lines. The inferred migration pathway represents the minimum distance traveled between two (or more) detection points and is thus a simplification of the actual route taken. The movement between successive points was determined for each post‐smolt using the *RunResidence* function in the *VTrack* package in R (Campbell, 2013). Migratory pathways were then mapped on to the Irish Sea and seas to the west of Scotland using the QGIS v.3.14 function, Points to Paths (https://qgis.org/en/site/). In instances where a post‐smolt passed a monitoring line undetected but was detected on a subsequent monitoring line, the receiver on the previous array that detected the largest number of fish was used as a surrogate of its position there for illustrative purposes. The determined migratory pathways of post‐smolts were grouped into nine regions comprising river systems draining into common coastal areas. The regions are as follows: Region 1, the Solway Firth; Region 2, the Clyde Sea; Region 3, Loch Linnhe; Region 4, Loch Torridon; Region 5, Loch Eireasort; Region 6, Loch Laxford; Region 7, Bush coastal region; Region 8, Lough Foyle; Region 9, Clew Bay; Region 10, east coast Ireland (more details in Figure 1b).

### 2.6 Detection frequency

The detection frequency was defined as the number of post‐smolts detected on a marine array as a proportion of the total number that entered the study area (i.e., detected leaving the river system, sea loch, or coastal embayment, depending on population) within the manufacturer‐provided lifetime of the tags used (typically 100–129 days). This was calculated for each monitoring line included in the study.

### 2.7 Migration duration and speed

Migration duration was calculated as the elapsed time between the final detection of a tag at one monitoring point or line and the first detection at a subsequent monitoring point or line.

Speed of migration (or rate of movement) was determined as the minimum (straight line) distance through marine waters between one monitoring point or line and the next (the migration pathway) divided by the time elapsed between last and first detections between detection points, expressed as both body lengths per second (L_F_ · s^−1^) and kilometers per day (km · day^−1^).

## 3 RESULTS

The mean (± SD) fork length of all smolts tagged in this study combined was 151.3 ± 19.4 mm and the mean weight was 37 ± 19.3 g. The mean tag burden was 0.05 ± 0.02 (Table 1).

In total, 1105 smolts were detected entering the study area (57.7% of the total number tagged) (Table 1). For rivers draining into estuaries and coastal embayment (i.e., the rivers Endrick, Gryffe, Bush, Faughan, Roe, and Burrishoole), 69.2% of tagged smolts that exited the river systems were detected exiting the estuaries or coastal embayments to reach coastal waters (Tables 4 and S3). Where it could be determined, the percentage of post‐smolts detected exiting coastal embayments and estuaries varied markedly. For example, 53.2% of post‐smolts were detected exiting Lough Foyle, whereas 87.7% of post‐smolts were detected exiting Runkerry Bay (Table 4).

**TABLE 4:** A description of the estuaries and coastal embayments included in this study, as well as mean distance (in kilometers) between the riverine receiver at the river mouth at the exit point of the estuary/embayment, the percentage and number of fish detected leaving the estuary/embayment, the migration speed (km · day^−1^), and duration (days) of passage through the estuary and embayment.

Tidal coastal inlet	Description of inlet	Mean distance (km)	% (no.) of fish detected exiting	Mean migration speed (km · day^−1^) ± SD	Mean duration (days) ± SD
Clyde Estuary	Extended estuary	52.6	82.5 (118)	13.66 ± 5.66	5.04 ± 2.87
Runkerry Bay	Open tidal embayment	1.8	87.7 (64)	35.22 ± 30.75	0.19 ± 0.36
Lough Foyle	Estuary	22.4	53.2 (25)	11.95 ± 5.64	2.65 ± 1.96
Clew Bay	Sheltered tidal embayment	20.2	76.5 (52)	29.93 ± 16.71	1.33 ± 1.88

*Note*: For detailed information see Table S3.

**FIGURE 2:**
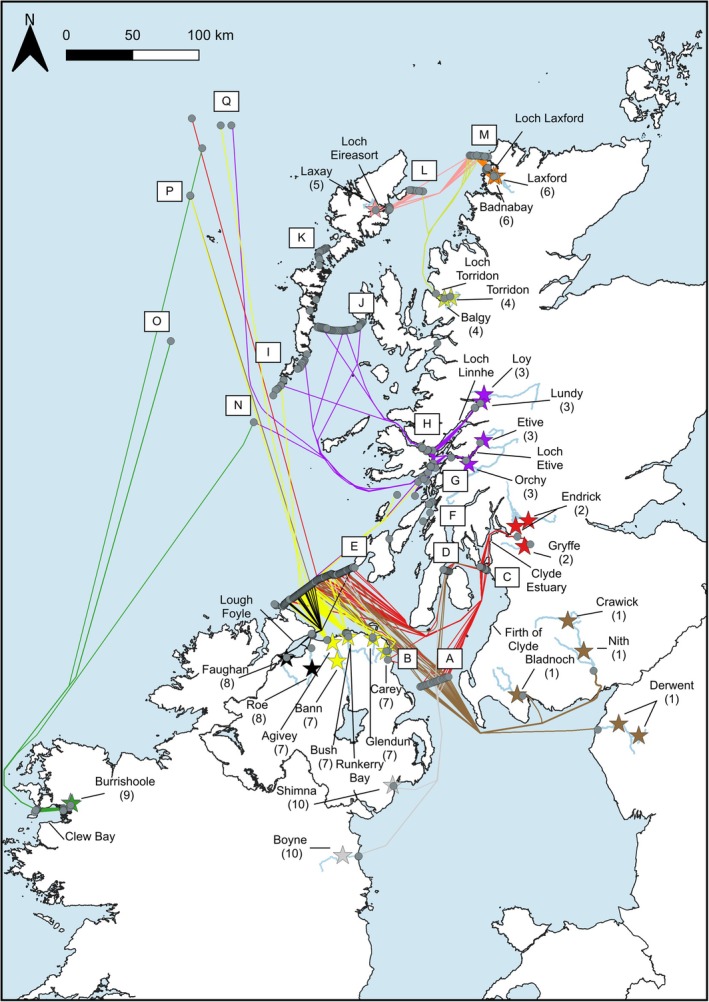
Map illustrating the estimated migratory pathways of Atlantic salmon post‐smolts as they migrate from their natal rivers (*n* = 25) in Scotland, England, Northern Ireland, and Ireland from detections on monitoring points/lines A–Q (see Figure 1 and Table 3 for more detail). The pathways of post‐smolts from each region are represented by a unique color, and each line represents an individual post‐smolts pathway. The regions are as follows: Region 1, the Solway Firth (rivers Derwent, Nith, and Bladnoch); Region 2, the Clyde Sea (rivers Endrick and Gryffe); Region 3, Loch Linnhe (rivers Etive, Orchy, and Lochy); Region 4, Loch Torridon (rivers Balgy and Torridon); Region 5, Loch Eireasort (River Laxay); Region 6, Loch Laxford (rivers Laxford and Badnabay); Region 7, Bush coastal region (rivers Bann, Bush, Carey, and Glendun); Region 8, Lough Foyle (rivers Roe and Faughan); Region 9, Clew Bay (River Burrishole); Region 10 (rivers Boyne and Shimna). The pathways illustrated are simplified representations and do not represent the true migratory pathways post‐smolts undertook.

On reaching marine waters, 39.6% (*n* = 438) of the post‐smolts that entered the study area (*n* = 1105) were detected on at least one marine monitoring line or point (Table 5). A total of 16.2% (*n* = 137) of post‐smolts entering the study area from Regions 1, 2, 3, 7, 8, and 10 (Figure 2) were detected on monitoring line E. A further 35.1% (*n* = 152) of post‐smolts entering the study from Regions 3 and 7 were detected at monitoring lines G and H. A smaller percentage (11%, *n* = 13) of post‐smolts that entered the study from Regions 4 and 5 was detected at monitoring line L. Finally, 25.5% (*n* = 47) of post‐smolts entering the study from Regions 4, 5, and 6 (total *n* = 184) were detected at monitoring line M (Table 5). Due to monitoring lines being deployed in marine waters, the detection range and efficiency of these lines would be expected to vary with local environmental conditions throughout the study. In addition, monitoring lines did not always cover the entire channel; therefore, these are minimum estimates of the percentage of post‐smolts detected passing each monitoring line.

**TABLE 5:** Summary statistics for each monitoring line and point. The number of post‐smolts detected on each monitoring line/point (the number used to determine migration speed), the region of origin of those post‐smolts, the range of dates that post‐smolts were detected and the mean migration speed of post‐smolts as they migrate to that monitoring line/point from the river mouth or tidal coastal inlet exit expressed in body lengths per second (LF · s^−1^) and in km · day^−1^ ± SD and (range). Rivers Endrick and Gryffe smolts were excluded from monitoring line A calculation as this served as their exit from their natal estuary.

Monitoring line/point	No. detected on array	Regions of origin	Date range detected	Mean migration speed (LF s^−1^) ± SD (range)	Mean migration speed (km · day^−1^) ± SD (range)
A	47 (45)	1, 2, & 10	06‐05 to 06‐06	0.94 ± 0.48 (0.18–2.01)	11.83 ± 5.87 (2.38–25.34)
B	1 (1)	2	15‐05	0.40	6.77
C	1 (1)	2	11‐06	0.72	9.10
D	8 (6)	1, 2, & 10	28‐04 to 10‐06	0.58 ± 0.33 (0.16–1.04)	7.72 ± 4.8 (2.05–15.93)
E	135 (135)	1, 2, 3, 7, 8, & 10	21‐04 to 20‐06	1.74 ± 0.98 (0.33–4.67)	23.57 ± 13.93 (3.87–61.64)
G	81 (72)	3 & 7	23‐04 to 21‐07	1.47 ± 0.68 (0.38–3.36)	17.47 ± 7.89 (4.29–38.05)
H	71 (62)	3	21‐04 to 20‐07	1.86 ± 0.83 (0.56–4.18)	22.05 ± 9.64 (6.43–46.96)
I	3 (1)	3 & 7	11‐05 to 05‐06	1	11.62
J	4 (3)	3	02‐05 to 27‐05	1.20 ± 0.47 (0.73–1.68)	14.40 ± 4.99 (9.61–19.56)
L	13 (7)	4 & 5	22‐04 to 17‐05	1.41 ± 0.38 (0.63–1.72)	16.93 ± 5.32 (7.20–21.19)
M	47 (40)	4, 5, & 6	01‐05 to 17‐05	1.93 ± 1.05 (0.46–4.44)	23.89 ± 13.06 (6.75–53.38)
N	2 (1)	3 & 9	27‐05	1.11	13.79
O	2 (2)	9	08‐06	3.03 ± 4.04 (0.18–5.89)	64.31 ± 85.36 (3.95–124.67)
P	2 (1)	1 & 9	19‐05 to 08‐06	1.93	25.87
Q	4 (3)	2, 3, 7, & 9	23‐05 to 06‐04	1.56	20.92

### 3.1 Migration pathways

**FIGURE 3:**
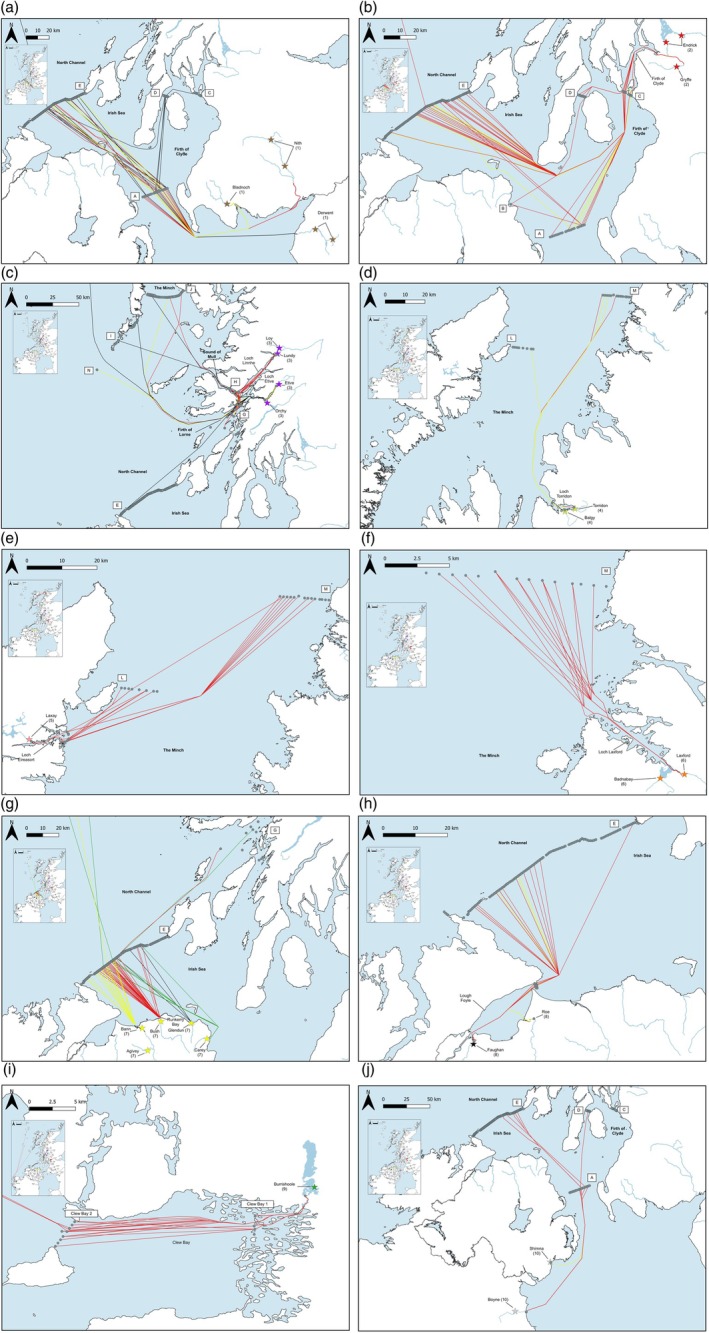
Maps illustrating the migratory pathways of Atlantic salmon post‐smolts as they migrate from their natal rivers to sea. The pathways illustrated are simplified representations and do not represent the true migratory pathways post‐smolts undertook. Migratory pathways were grouped into eight monitoring regions based on rivers that drain into the same coastal environment. These include (a) Region 1: Solway Firth (rivers Derwent, Nith, Crawick, and Bladnoch); (b) Region 2: Clyde marine region (rivers Endrick and Gryffe); (c) Region 3: Loch Linnhe (rivers Loy, Lundy, Etive, and Orchy); (d) Region 4: Loch Torridon (rivers Torridon and Balgy); (e) Region 5: Loch Eireasort (River Laxay); (f) Region 6: Loch Laxford (rivers Laxford and Badnabay); (g) Region 7: Bush marine region (rivers Bann, Agivey, Bush, Carey, and Glendun); (h) Region 8: Foyle marine region (rivers Roe and Faughan); (i) Region 9 (River Burrishole); and (j) Region 10 (rivers Boyne and Shimna). The pathways of post‐smolts from each river system are given a unique color, and each line represents an individual post‐smolt pathway.

Atlantic salmon post‐smolts migrated in multiple and complex directions through inshore and offshore waters. These migration pathways are summarized below for each of the regions included in this study (Figures 2 and 3).

#### 3.1.1 Region 1: Rivers Derwent, Nith, and Bladnoch (*N* = 184; Figure 3a)

Salmon post‐smolts (*N* = 69; 37.5%) from all four rivers in this region were detected on two of the most southerly monitoring lines (monitoring lines A and E of Figure 3a). Post‐smolts from Region 1 were detected on monitoring line A between May 11 and June 6 and on monitoring line E between May 13 and June 20. Interestingly, four post‐smolts (2.2%) from the River Derwent, England, were detected within the Firth of Clyde on monitoring lines C and D between June 6 and July 13. Of these four post‐smolts, one was subsequently detected leaving the Firth of Clyde (Figure 1a) and detected on monitoring line E. Finally, one post‐smolt (0.5%) from the River Derwent was detected to the west of the Hebrides at monitoring point P on June 8, 575 km from its natal river.

#### 3.1.2 Region 2: Rivers Endrick and Gryffe (*N* = 143; Figure 3b)

Salmon post‐smolts from the rivers Endrick and Gryffe exited the Clyde Estuary and Firth of Clyde utilizing multiple routes (Figure 3b). Post‐smolts migrated both east (*N* = 5; 3.5%) and west (*N* = 113; 79.0%) around the island of Little Cumbrae (monitoring line C), as well as west of the island of Arran (*N* = 4; 2.8%) (monitoring line D) (Figure 1a) and east (*N* = 39; 27.3%), assumed to have traveled east around the island if not detected on monitoring line D but detected on subsequent monitoring lines around Arran (monitoring line D). Once post‐smolts left the Firth of Clyde, 36 (25.2%) were detected on monitoring line E between May 4 and June 18. A small number (*N* = 10; 7.0%) of post‐smolts migrated south and were detected on monitoring line A between May 6 and June 4. Three of these post‐smolts subsequently migrated north and were detected on monitoring line E. The remaining seven post‐smolts were not detected again. One post‐smolt (0.1%) from the River Gryffe was detected approximately 548 km from its natal river at the continental shelf by the Slocum glider (AUV) (monitoring point Q) on May 23. Finally, one post‐smolt (0.1%) from the River Gryffe left the Firth of Clyde and migrated west to be detected in a coastal embayment (monitoring point B) in Northern Ireland on May 15. This post‐smolt was then detected on monitoring line A on June 4 before migrating north again to be detected at monitoring point E on June 6.

#### 3.1.3 Region 3: Rivers Loy, Lundy, Etive, and Orchy (*N* = 251; Figure 3c)

Salmon post‐smolts from Region 3 leaving Loch Linnhe and Loch Etive could migrate either through the Firth of Lorne (monitoring line G) or the Sound of Mull (monitoring line H) (Figures 1a and 3c). Post‐smolts from this region utilized both of these routes and were detected in the Firth of Lorne (*N* = 79; 31.5%) between April 23 and July 21 and in the Sound of Mull (*N* = 71; 28.3%) between April 21 and July 20. Four post‐smolts (1.6%) were detected on monitoring line J between May 2 and 27, thus, appearing to pass through the Minch (the waters between mainland Scotland and the outer Hebridean islands); two (0.8%) were detected in the waters between the southern Hebridean islands (monitoring line I) between May 11 and 13; one post‐smolt (0.4%) migrated south to be detected at monitoring line E on May 15; and one post‐smolt (0.4%) was detected at monitoring point N on May 27. Finally, one post‐smolt (0.4%) from the River Orchy was detected at monitoring point Q around 100 km to the west of the Isle of Lewis and approximately 362 km from its natal river on May 29.

#### 3.1.4 Region 4: Rivers Balgy and Torridon (*N* = 54; Figure 3d)

Salmon post‐smolts from Region 4 left Loch Torridon and were detected on two monitoring lines (Figure 3d). Post‐smolts were detected on monitoring line L off the east coast of the Isle of Lewis (*N* = 1; 1.9%) (Figure 1a) on May 1 and on monitoring line M off the west coast of the northern tip of mainland Scotland (*N* = 6; 11.1%) between May 6 and 13.

#### 3.1.5 Region 5: River Laxay (*N* = 64; Figure 3e)

Salmon post‐smolts from Region 5 left Loch Eireasort and were detected on two monitoring lines (Figure 3e). Post‐smolts were detected on monitoring line L off the east coast of the Isle of Lewis (*N* = 12; 18.8%) (Figure 1a) between April 22 and May 17 and on monitoring line M off the west coast of the northern tip of mainland Scotland (*N* = 13; 20.3%) between May 2 and 17.

#### 3.1.6 Region 6: Rivers Laxford and Badnabay (*N* = 66; Figure 3f)

Post‐smolts from Region 6 were detected on monitoring line M of the northwest coast of mainland Scotland (*N* = 29; 43.9%) between May 1 and 17 (Figure 3f).

#### 3.1.7 Region 7: Rivers Bann, Agivey, Bush, Carey, and Glendun (*N* = 135; Figure 3g)

Post‐smolts from Region 7 were detected on monitoring line E between Ireland and Scotland (*N* = 68; 50.4%) between May 2 and 29 (Figure 3g). One post‐smolt (0.7%) from the River Glendun was detected between the southern Hebridean islands (monitoring line I) on June 5 (Figure 1a). Two post‐smolts (rivers Glendun and Bush; 1.5%) migrated east and were detected in the Firth of Lorne (monitoring line G) between April 28 and May 26. One post‐smolt (0.7%) from the River Glendun was detected at monitoring point O on May 19. Finally, one post‐smolt (0.7%) from the River Bann was detected approximately 402 km from its natal river by the Slocum glider (AUV) at monitoring point Q on May 31.

#### 3.1.8 Region 8: Rivers Faughan and Roe (*N* = 47; Figure 3h)

Salmon post‐smolts from Region 8 (*N* = 23; 48.9%) were detected on monitoring line E between May 6 and June 1 (Figure 3h).

#### 3.1.9 Region 9: River Burrishoole (*N* = 74; Figure 3i)

Post‐smolts from Region 9 were detected at several marine monitoring points, which included monitoring points N (*N* = 1; 1.4%), O (*N* = 2; 2.7%), and Q (*N* = 1; 1.4%) to the south and west of the Hebrides, on May 24, June 5, and June 4, respectively (Figures 1a and 3i). All post‐smolts detected at marine monitoring points were of ranched origin.

#### 3.1.10 Region 10: Rivers Boyne and Shimna (*N* = 87; Figure 3j)

Post‐smolts from Region 10 (*N* = 14; 16.1%) were detected on three arrays in the Irish Sea (Figure 3j). They were detected on monitoring line A (*N* = 13; 14.9%) between May 15 and June 3, monitoring line D (*N* = 1; 1.1%) on May 28, and monitoring line E (*N* = 6; 6.9%) between May 20 and June 10.

### 3.2 Speed and duration of migration

The mean migration speed of post‐smolts through coastal embayments and estuaries differed. The mean migration speed varied from 0.06 L_F_ · s^−1^ (4.41 km · day^−1^) for River Roe post‐smolts migrating through Lough Foyle (Region 8) to 2.43 L_F_ · s^−1^ (35.22 km · day^−1^) for River Bush post‐smolts migrating through the open Runkerry Bay (Region 7) (Tables 4 and S3).

**FIGURE 4:**
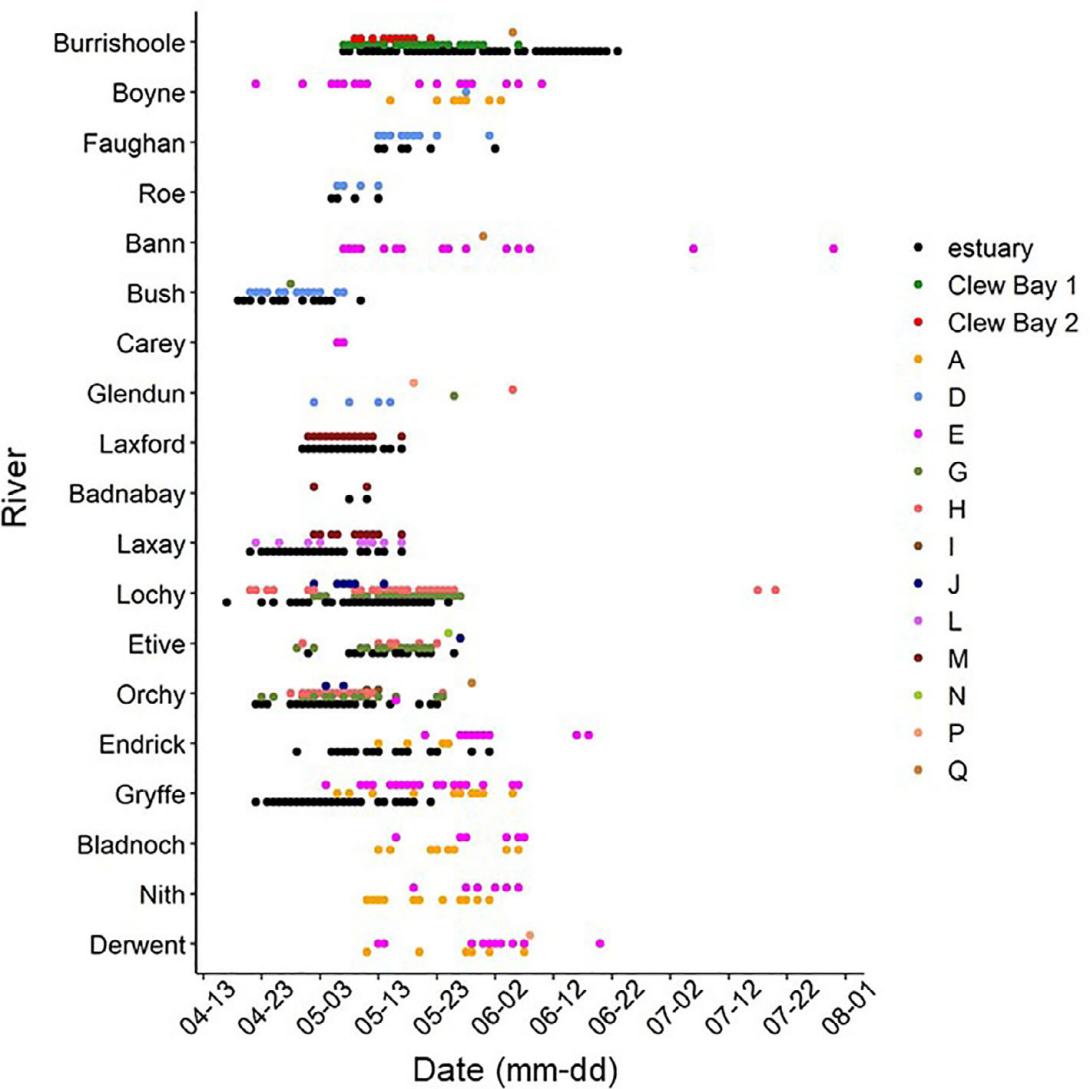
Abacus plot displaying dates (mm‐dd) when acoustically tagged Atlantic salmon post‐smolts (*n* = 1105) were detected at monitoring points/lines in this study (see methods; Figure 1). This included monitoring lines deployed in tidal coastal inlets as well as monitoring points/lines A–Q (Figure 1).

The duration of the migration through coastal embayments and estuaries also varied, with the shortest mean duration (±SD) being 0.19 ± 0.36 days through the 1.81 km of Runkerry Bay and the longest mean duration being 5.04 ± 2.87 days through 6.60 km of Clew Bay (Figures 1a and 4; Table 4).

Once post‐smolts entered inshore coastal waters, their mean migration speed increased but also varied between regions. The mean migration speed varied from 0.34 L_F_ · s^−1^ (4.32 km · day^−1^) for a post‐smolt from the River Gryffe migrating to monitoring line D to 3.08 L_F_ · s^−1^ (39.94 km · day^−1^) for River Roe post‐smolts migrating to monitoring line E (Tables 5 and S3; Figure S1).

The time taken to reach monitoring lines varied among rivers and regions. For example, it took post‐smolts from the River Lochy a mean of 12.74 days to migrate 203 km to monitoring line J, whereas it took post‐smolts from the River Etive 14.16 days to migrate 172 km to monitoring line J. Furthermore, post‐smolts from the River Glendun took, on average, 2.52 days and post‐smolts from the River Bann took 1.31 days to migrate to monitoring line E despite covering similar distances (47.1 and 45.6 km, respectively) (Tables 5 and S3; Figure S1).

In this study, the longest distance over which a post‐smolt was tracked was 564 km. This fish originated from the River Burrishoole (Table S3) and took approximately 26.09 days to migrate from leaving Clew Bay to monitoring point Q (Table S3).

## 4 DISCUSSION

The principal aim of this study was to provide empirical data to elucidate the movement patterns and pathways of post‐smolt Atlantic salmon migrating from the west of Scotland, Ireland, northwest England, and Northern Ireland through coastal waters and offshore marine waters. By using data from 25 populations from four jurisdictions across a single year, this study provides insights on spatial and population variation in migration pathways across a broad geographic scale. Alternative approaches to address the same questions include ecological modeling of migration that has resulted in useful working hypotheses that require testing with empirical data. Trawling studies have provided informative empirical data on marine distribution, but these lack positional precision and can only provide insights into migration behavior at a very broad scale with little definition.

The demanding logistics and very high costs of telemetry studies have, until now, been limited to single populations, small sample sizes, and very restricted spatial coverage in the marine environment (Barry et al., 2020; Ounsley et al., 2020; Gilbey et al., 2021; Green et al., 2022). This study has partly circumvented some of the very considerable logistical challenges of telemetry studies on fish by pooling resources and data from 10 projects that operated 17 monitoring locations in marine waters in 2021. The geographic range and the sample sizes provided by this study provide insights that would not result from a single population, narrow geographic range approach. Here the maximum migration duration and inferred distance of post‐smolts tracked in this study were approximately 100 days and 575 km, respectively. These data provide an unprecedented insight into the use of coastal zones by sea‐migrating salmon post‐smolts from across a broad geographic region (approximately 107,620 km^2^) in Europe.

### 4.1 Migration pathways

This study has allowed for the first empirical description of salmon post‐smolt migration pathways in inshore (and to a lesser extent offshore) coastal waters to the west of the British Isles. We demonstrate high levels of within‐ and between‐river variation in the routes taken through marine waters from the mouth of their natal rivers toward the continental shelf edge.

Overall, post‐smolts from rivers draining into the Solway, Clyde, Boyne, Bush, and Foyle marine areas (Regions 1, 2, 7, 8, and 10) tended to migrate in a northerly direction, being detected passing through the North Channel at the northern end of the Irish Sea. Similarly, the detection of a fish from Clew Bay on the Irish west coast (Region 9) to the west of the Hebrides (Figure 1A) suggests that fish from this location were also migrating north (Figure 2). Future studies using predator tags could help determine the nature of these types of behaviors. After leaving the Irish Sea (monitoring line E), most fish were not detected again, except for six post‐smolts from the Solway, Clyde, and Foyle (Regions 1, 2, and 8). Five of these were detected to the south and west of the island chain comprising the Hebrides (monitoring lines and points I, O, P, and Q; Figures 1A and 2). The sixth fish was detected south of the island of Mull and thus appears to have migrated to the northeast. No fish from rivers draining into the Solway, Clyde, Bush, or Foyle (Regions 1, 2, 7, and 8) nor fish from Clew Bay on the Irish west coast (Region 9) were detected in the channel separating the Outer Hebrides from the Scottish western mainland (the Minch) (i.e., at J, L, or M, Figures 1a and 2). This suggests that once post‐smolts left the Irish Sea, they did not migrate through the Minch waters (between mainland Scotland and the Outer Hebrides) but most likely migrated broadly west in the waters between the Hebridean islands and the island of Ireland. This finding provides some empirical support for the northwest swimming behavior model for migration of salmon post‐smolts from populations in southwest Scotland (and presumably northwest England) proposed by Ounsley et al., 2020. These data similarly indicate that post‐smolts from the Clew Bay (from the River Burrishoole; Region 9) also did not migrate through the Minch.

This possibility is further supported by data from salmon post‐smolts emanating from rivers draining into the Loch Linnhe marine area (Region 3). Here only 4 out of the 150 salmon post‐smolts that left the waters around the island of Mull were detected migrating through the Minch, with a further 2 migrating between the southern islands of the Outer Hebrides (Figures 1a and 2). It is possible that the majority of post‐smolts from this region migrated west in the waters between the south of the Hebrides and the island of Ireland, which is further supported by the detection of a single post‐smolt from the Loch Etive area on the AUV operating on the continental shelf edge to the west of the Hebrides (at Q; Figure 2). Therefore, this possibility would merit further investigation. Moriarty et al. (2023) showed that post‐smolts passing through the Minch could be expected to acquire an increased load of the parasitic sea louse *Lepeoptherius salmonis* emanating from salmon farming units, which are relatively more dense in this area. One working conclusion from the pathway information in this study is that the general risk of exposure to sea louse infection likely differs between populations, with populations from rivers draining into the Solway, Clyde, Foyle, and Bush marine areas likely to be less exposed to the risk of infection than post‐smolts from the other regions examined here (Linnhe, Torridon, Eireasort, and Laxford), where post‐smolts do migrate through the Minch.

This study found between‐individual, within‐population variation in migration route. For example, fish from the rivers Derwent (Region 1), Endrick/Gryffe (Region 2), Boyne (Region 10), and Orchy (Region 3) adopted several different migratory pathways. Detections of post‐smolts from the River Orchy indicated multiple migration pathways, including through the Minch waters (at monitoring line J; Figures 1a and 2), between the southern islands of the Outer Hebrides (at monitoring line I; Figure 2), south toward Northern Ireland (at monitoring line E; Figure 2), and west toward the continental shelf (at monitoring line Q; Figure 2). All of these detections are consistent with a migration to the north and west that would be expected of a migrating salmon post‐smolt and, therefore, a logical conclusion is that post‐smolts from this population are using multiple migration pathways through the coastal areas into the eastern North Atlantic.

Post‐smolts from the rivers Endrick and Gryffe tended to migrate in a northerly direction through the Irish Sea; however, seven post‐smolts from this region (Region 2) were initially detected migrating in a southerly direction (detected on monitoring line A) (Figure 2), after which three of these post‐smolts were subsequently detected migrating north, out through the North Channel (monitoring line E; Figures 1a and 2). In addition, to this unexpected behavior, four fish from the River Derwent and one from the River Boyne migrated into the Clyde Estuary (detected at monitoring lines C and D; Figure 2), deviating from the expected, most direct northerly migratory trajectory. Of the five fish that entered the Clyde Estuary (from the Derwent and Boyne), one exited the estuary and was later detected leaving the Irish Sea via the North Channel (detected at monitoring line E; Figure 2). There are two explanations as to why these unexpected movement patterns could have occurred. First, these post‐smolts may have been diverted by coastal flows. For example, the southerly coastal flows generated by the high volume of freshwater input from the Clyde Sea that extends toward the Mull of Galloway may explain why post‐smolts from Region 2 migrated south through the Irish Sea (Kasai et al., 1999; Young et al., 2000). Second, these fish could have been predated, and the behavior observed is that of predators. However, a proportion of these post‐smolts were subsequently detected exiting the Irish Sea via the North Channel (detected at monitoring line E; Figure 2), suggesting that (at least for these fish) these are detections of a migrating post‐smolt rather than detections of a tag inside a predator.

A proportion of smolts that entered the study area were not detected on any of the monitoring lines or points included in this study. There are several possible explanations for this. Therefore, post‐smolts could potentially have migrated through an area of sea not covered by monitoring lines and points deployed during this study. Tag failure is another possibility. Post‐smolts may have migrated passed monitoring lines undetected, or post‐smolts may have been subject to predation (Thorstad, Uglem et al., 2012). The use of predation tags in future studies may help with the interpretation of outlier behavior and quantify the proportion of post‐smolts that are predated in inshore and offshore waters (Buchanan & Whitlock, 2022; Lennox et al., 2023).

Despite the broad geographic coverage and multiple salmon populations covered in this study, it was conducted only over a single seasonal migration. Therefore, we are unable to determine if the considerable spatial variation in migration pathways described here is also matched with similar inter‐year temporal variation. Future studies are needed to examine this.

Trawling studies have shown that once they leave their natal rivers, post‐smolts from the UK and Ireland migrate toward the continental shelf edge, west of the Hebridean islands, where there is a relatively strong current, north easterly current (Gilbey et al., 2021). The particle tracking study by Ounsley et al. (2020) indicated that post‐smolts emanating from rivers on the west coast of Scotland could not rely solely on current following but would need to actively swim to reach the continental shelf edge. This same study also concluded that region or population‐specific migration tactics would be needed for fish from different natal rivers to make successful migrations out from the coastal zones (Ounsley et al., 2020). The empirical pathway results from the study presented here broadly support this conclusion, showing that fish from different regions do indeed adopt different migration pathways (Figure 2). However, here we also show that there is considerable within‐population between‐individual variation in migration pathway choice. The basis for this individual variation is not yet clear. However, it is interesting to compare the interpopulation and interindividual variation in migration pathways taken by post‐smolts with that of large adult fish that had previously spawned and were subsequently returning to feeding areas in the open ocean. Although over a much wider geographic range, a recent study by Rikardsen et al. (2021) on previously spawned fish showed very considerable variation in migration pathway between populations and individuals across the whole of the North Atlantic, suggesting that fish from different regions adopted different pathways and used different foraging areas at sea.

### 4.2 Speed and duration of migration

Similar to other studies, we found that salmon post‐smolts spent variable periods of time migrating through estuaries and coastal embayments, with the difference primarily being driven by the variation in basin shapes (Chaput et al., 2019; Dempson et al., 2011; Kocik et al., 2009; Thorstad et al. 2012a). Post‐smolts took on average 0.19 days to migrate through Runkerry Bay, whereas the mean was 5.18 days to migrate through the Clyde Estuary (Table 4). Variation in migration time through coastal waters was also evident, but it primarily reflects variation in travel distances by post‐smolts. However, migration duration did, in some instances, vary independently of distance traveled. For example, post‐smolts from the rivers Bladnoch and Nith took on average 2.7 and 3.6 days, respectively, to travel through the same area of the Irish Sea (Table S3; Figure S1).

In this study, the mean speed of post‐smolts migrating through estuaries and coastal embayments ranged from 5.4 km · day^−1^ for River Roe post‐smolts migrating through Lough Foyle to 35.2 km · day^−1^ for River Bush post‐smolts migrating through Runkerry Bay (Table S3). The mean speed of post‐smolts migrating through coastal waters was much higher than those migrating through estuaries and coastal embayments and ranged from 4.32 to 53.8 km · day^−1^ (Table S3; Figure S1). Previous studies have reported similar ranges in migratory speeds in estuarine and marine waters (Halfyard et al., 2012; Lacroix, 2008; Lefèvre et al., 2013; Lilly et al., 2022; Lothian et al., 2018; Stich et al., 2015). Migration speeds have also been shown to increase as post‐smolts migrate from coastal embayments and estuaries toward coastal waters (Davidsen et al., 2009). Considerable variation was shown in the migration speed between water bodies and individual fish. For example, the mean migration speed for post‐smolts migrating between the Firth of Clyde (monitoring line C) and the Irish Sea (monitoring line E) ranged from 7.11 to 11.95 km · day^−1^ and from 4.41 to 28.84 km · day^−1^ for rivers Endrick and Gryffe, respectively. The considerable variation in migration speed of post‐smolts between water bodies, rivers, and individuals could be driven by a number of factors, including smolt size, current speed and direction, as well as simply due to individual behavior (Davidsen et al., 2009; Doogan et al., 2023; Newton et al., 2021).

### 4.3 Pressures and management implications

The migration pathways indicated in this study showed that salmon post‐smolts are migrating through multiple legislative jurisdictions once at sea. As knowledge of these pathways develops, this will allow us to make spatially explicit linkages between migration pathways and putative pressures. The main threats to Atlantic salmon in the inshore and nearshore coastal marine environment, including predation, fisheries by‐catch, aquaculture, and offshore renewables (Scottish Government, 2022), have been thoroughly reviewed recently elsewhere (Gillson et al., 2022) and so are not reiterated here in detail. Each of these putative impacts is non‐randomly distributed and is mostly concentrated in space and/or time in the coastal marine environment. In some places, a specific pressure may overlap with another spatially or temporally, potentially resulting in additive effects of impact. For other pressures, the impact may occur at a considerable distance from the undertaken activity (e.g., parasitic infection risk with distance from aquaculture units; Scanlon et al., 2021). The cross‐legislative boundary nature of the migration pathways of salmon post‐smolts compounds the complexity of management of the species. Although beyond the scope of the study presented here, a logical and important next step is to combine migration pathway and timing information with spatial, temporal, and effect size data on potential salmon population stressors to quantify risk to migrating salmon smolts and post‐smolts.

The broad spatial extent and large sample size achieved in the study presented here demonstrate the value of cross‐organization and cross‐jurisdictional collaboration. The collaboration that has resulted in this study has enabled insights into migration patterns of this highly mobile, migratory species that would be unlikely from a single project. Indeed, the transboundary migration pathways of post‐smolts observed here emphasize the importance of increasing such knowledge to better inform the formulation of policy and management actions at the international level. This broad‐scale acoustic telemetry study would not have been possible without extensive collaboration between a very large number of individual scientists, organizations, and projects. The financial, logistic, and resource costs of conducting a telemetry project over a wide geographic area are high and become affordable and realistic only if shared. It is estimated that the combined cost of the projects included in this study was in the region of £2.94 million and required a team of approximately 70 individuals. In addition to this, the data sharing for this collaboration was only possible because of the technical compatibility of tags and receivers from the multiple equipment suppliers. This illustrates the vital importance of such commonality to capitalize fully on the potential of telemetry studies at an international scale.

## 5 CONCLUSION

This study provides valuable empirical information on the migration pathways used by Atlantic salmon post‐smolts migrating from four separate countries (Scotland, England, Northern Ireland, and Ireland), each with separate species protection legislation and contrasting management policies, many of which will have potential for significant impact on the migration success for this species. We demonstrate considerable between‐ and within‐river variation in migration pathways adopted by fish migrating through inshore and offshore marine waters in these jurisdictions during one migration season. A logical inference from this is that some populations and individuals are likely at more risk from known natural and anthropogenic environmental pressures than others. Important future steps would include linking these data with the spatial distribution of known pressures to assess the magnitude of that risk for different populations, as well as determining the extent and nature of temporal variation in migration pathways of salmon post‐smolts.

## AUTHOR CONTRIBUTIONS

JRR, JL, HMH & CEA designed and planned this study which collates the data collected by several acoustic telemetry projects. All authors contributed to the planning, design and fielwork for these projects. JL and JRR analysed the data. JRR, JL & CEA drafted the manuscript with feedback from all other authors.

## FUNDING INFORMATION

This study was funded by Maritime Fisheries Fund, Salmon Scotland, the Atlantic Salmon Trust, EU award IVA5060 from the Interreg 5A programme, EU Interreg 5A managed by the Special EU Programmes body funded COMPASS*, Environment Agency, Natural England, The Derwent Owners Association, United Utilities PLC, NatureScot, the Nith District Salmon Fishery Board, Holywood Trust, and Dumfries and Galloway Council.* The views and opinions expressed in this document do not necessarily reflect those of the European Commission or the Special EU Programmes Body (SEUPB).

## Supporting information


**Figure S1.** Boxplots displaying the total duration (days) and migration speed between monitoring lines (kilometers per day [km · day^−1^]) (only for rivers where more than one smolt was detected on the monitoring line).

**Table S1.** Table describing the objectives of the seven projects that tagged salmonid fish and/or deployed fixed receivers operating on 69 kHz that allowed reciprocal detection of fish and three projects where receivers operating on compatible 69 kHz were deployed for other reasons but from which fish detections were shared.
**Table S2.** The total number of acoustic receivers deployed and retrieved, as well as the duration they were deployed during 2021. Four types of acoustic receivers were deployed in this study, including VR2W, VR2Tx, VR2AW, and TR700, a basic description of each receiver type can be found in Lilly et al. (2021) and at https://www.thelmabiotel.com/receivers/tbr-700/. This table expands on the data presented in a sister paper (Lilly et al., 2023).
**Table S3.** The total number of Atlantic salmon post‐smolts detected at key monitoring points/lines in this study. The statistics calculated in this table were calculated for each section of the migratory journey, which included the number of post‐smolts detected at the start and end arrays, and the date range post‐smolts were detected on the end array for each section. The total duration (days) and rate of movement (ROM) between monitoring lines (body length per second [L_F_ · s^−1^]/kilometers per day [km · day^−1^]) were also calculated. There were some instances where not all smolts detected on the end array were also detected on the start array. Therefore, in these cases ROM was calculated using a proportion of the smolts detected. Est, estuary; CE, coastal embayment. This table expands on the data presented in a sister paper (Lilly et al., 2023).
