# Supplementary material for: Inshore and offshore marine migration pathways of Atlantic salmon post‐smolts from multiple rivers in Scotland, England, Northern Ireland, and Ireland
Source: J Fish Biol. 2024 Apr 28;106(5):1422–39. doi: 10.1111/jfb.15760 (PMC12120341; doi:10.1111/jfb.15760)
Supplement: Supplementary file 2 — Table S1. Table describing the objectives of the seven projects that tagged salmonid fish and/or deployed fixed receivers operating on 69 kHz that allowed reciprocal detection of fish and three projects where receivers operating on compatible 69 kHz were deployed for other reasons but from which fish detections were shared. Table S2. The total number of acoustic receivers deployed and retrieved, as well as the duration they were deployed during 2021. Four types of acoustic receivers were deployed in this study, including VR2W, VR2Tx, VR2AW, and TR700, a basic description of each receiver type can be found in Lilly et al. (2021) and at https://www.thelmabiotel.com/receivers/tbr-700/. This table expands on the data presented in a sister paper (Lilly et al., 2023). Table S3. The total number of Atlantic salmon post‐smolts detected at key monitoring points/lines in this study. The statistics calculated in this table were calculated for each section of the migratory journey, which included the number of post‐smolts detected at the start and end arrays, and the date range post‐smolts were detected on the end array for each section. The total duration (days) and rate of movement (ROM) between monitoring lines (body length per second [LF · s−1]/kilometers per day [km · day−1]) were also calculated. There were some instances where not all smolts detected on the end array were also detected on the start array. Therefore, in these cases ROM was calculated using a proportion of the smolts detected. Est, estuary; CE, coastal embayment. This table expands on the data presented in a sister paper (Lilly et al., 2023). [file JFB-106-1422-s002.docx]

**Supplementary Material**

Table S1. Table describing the objectives of the seven projects that tagged salmonid fish and/or deployed fixed receivers operating on 69 kHz that allowed reciprocal detection of fish and three projects where receivers operating on compatible 69 kHz were deployed for other reasons but from which fish detections were shared.

| **Project** | **Project dates** | **Objectives for salmon tracking** | **Rivers included** |
| --- | --- | --- | --- |
| SeaMonitor Project | 2019 – 2022 | Determine salmon pathways, migration speed and loss rate at sea. | Multiple rivers in Scotland, Northern Ireland and Ireland |
| West Coast Tracking Project | 2020 – 2023 | Determine migration pathways of salmon from multiple catchments on the West of Scotland | Multiple rivers in Scotland |
| Nith Smolt Tracking Project | 2021 | Examining the freshwater migration of salmon smolts  Tracking pathways at sea | Crawick and Nith |
| Derwent Tracking Project | 2020 – 2022 | Tracking salmon in freshwater and coastal marine | Derwent |
| Burrishoole salmon tracking | Ongoing | Tracking salmonids at sea and in freshwater | Burrishoole |
| AFBI salmonid tracking project | Ongoing | Tracking salmonids in freshwater and at sea | Multiple catchments in Northern Ireland |
| COMPASS Project (Collaborative Oceanography & Monitoring for Protected Areas &Species) | 2017-2021 | Tracking salmonids at sea | Multiple catchments including Boyne and Shimna |
| Torridon Tracking Project | 2018 - present | To determine habitat usage and escapement timings of salmon and sea trout within Loch Torridon, Scotland | Balgy and Torridon |
| Movement ecology of flapper skate (MEFS) project | 2018 to present | Movement ecology of flapper skate (MEFS) (no aims around salmon telemetry) | 69 kHZ marine receiver deployment |
| Static Acoustic Monitoring of Scottish Atlantic Seas (SAMOSAS) | 2020-2021 | Passive acoustic monitoring of marine mammals in open seas (no aims around salmon telemetry) | 69kHZ marine receiver deployment |

Table S2. The total number of acoustic receivers deployed and retrieved, as well as the duration they were deployed during 2021. Four types of acoustic receivers were deployed in this study, including VR2W, VR2Tx, VR2AW and TR700, a basic description of each receiver type can be found in Lilly et al. (2021) and at https://www.thelmabiotel.com/receivers/tbr-700/. This table expands on the data presented in a sister paper (Lilly et al. 2023).

| **Type** | **Region** | **ID** | | **Number of receivers recovered (Number of receivers deployed)** | **Latitude** | **Longitude** | **Deployment Duration** | |
| --- | --- | --- | --- | --- | --- | --- | --- | --- |
| River | Leven | - | | 1 (1) | 55.939 | -4.563 | 16-03 to 05-07 | |
| River | Gryfe | - | | 1 (1) | 55.890 | -4.406 | 09-04 to 20-07 | |
| River | Bladnoch | - | | 1 (1) | 54.862 | -4.434 | 24-03 to 19-07 | |
| River | Nith | - | | 1 (1) | 55.046 | -3.607 | 06-04 to 01-07 | |
| River | Lochy | - | | 1 (1) | 56.832 | -5.085 | 25-03 to 01-07 | |
| River | Etive | - | | 1 (1) | 56.566 | -5.064 | 11-04 to 22-07 | |
| River | Orchy | - | | 1 (1) | 56.440 | -5.227 | 12-04 to 21-07 | |
| River | Laxford | - | | 1 (1) | 58.375 | -5.017 | 16-04 to 01-07 | |
| River | Badnabay | - | | 1 (1) | 58.372 | -5.045 | 23-04 to 01-07 | |
| River | Laxay | - | | 1 (1) | 58.099 | -6.535 | 30-03 to 01-07 | |
| River | Bush | - | | 1 (1) | 55.219 | -6.532 | 01-04 to 13-09 | |
| River | Glendun | - | | 1 (1) | 55.124 | -6.044 | 06-04 to 06-10 | |
| River | Bann | - | | 2 (2) | 55.171 | -6.773 | 07-04 to 04-08 | |
| River | Carey | - | | 1 (1) | 55.201 | -6.233 | 06-04 to 27-08 | |
| River | Derwent | - | | 1 (1) | 54.646 | -3.542 | 24-03 to 22-07 | |
| River | Roe | - | | 1 (1) | 55.109 | -6.951 | 26-04 to 20-06 | |
| River | Faughan | - | | 1 (1) | 55.035 | -7.226 | 26-04 to 20-06 | |
| River | Derwent | - | | 1 (1) | 54.646 | -3.542 | 24-03 to 22-07 | |
| River | Burrishoole | - | | 1 (1) | 53.906 | -9.580 | 04-05 to 14-06 | |
| River | Shimna | | - | 1 (1) | 54.211 | -5.891 | 01-01 to 31-12 |  |
| Estuary | Boyne | | - | 2 (2) | 53.722 | -6.246 | 01-01 to 31-12 |  |
| Estuary | Balgy | | - | 5 (5) | 57.531 | -5.596 | 13-04 to 05-10 |  |
| Estuary | Torridon | | - | 6 (6) | 57.540 | -5.515 | 01-04 to 23-09 |  |
| Estuary | Burrishoole | | - | 1 (1) | 53.887 | -9.588 | 04-05 to 14-06 |  |
| Estuary | Clyde Estuary | C | | 6 (6) | 55.725 | -5.000 | 02-04 to 06-08 | |
| Fjord | Firth of Clyde | D | | 6 (6) | 55.694 | -5.437 | 02-04 to 06-08 | |
| Sea Loch | Laxford | - | | 5 (5) | 58.424 | -5.141 | 12-04 to 30-08 | |
| Sea Loch | Laxay | - | | 6 (6) | 58.123 | -6.358 | 13-04 to 17-12 | |
| Sea Loch | Etive | - | | 2 (2) | 56.455 | -5.411 | 01-04 to 26-08 | |
| Sea Loch | Linnhe | - | | 2 (2) | 56.801 | -5.150 | 01-04 to 26-08 | |
| Coastal Embayment | Runkerry Bay | - | | 7 (7) | 55.226 | -6.557 | 03-04 to 13-09 | |
| Estuary | Lough Foyle | - | | 10 (10) | 55.202 | -6.948 | 24-03 to 20-06 | |
| Coastal Embayment | Waterfoot Bay | B | | 1 (1) | 55.064 | -6.041 | - | |
| Coastal Embayment | Clew Bay 1 | - | | 5 (5) | 53.871 | -9.656 | 28-04 to 22-06 | |
| Coastal Embayment | Clew Bay 2 | - | | 9 (10) | 53.843 | -9.964 | 28-04 to 22-06 | |
| Sea Loch | Loch Torridon | - | | 6 | 57.560 | -5.704 | 01-04 to 20-09 | |
| Marine | Mull (Sound of Mull) | H | | 7 (8) | 56.512 | -5.767 | 01-04 to 26-08 | |
| Marine | Mull (Sound of Lorne) | G | | 10 (12) | 56.383 | -5.620 | 31-03 to 26-08 | |
| Marine | Harris to North Uist | K | | 15 (15) | 57.791 | -7.201 | 02-04 to 22-08 | |
| Marine | North Uist to Benbecula | K | | 1 (1) | 57.466 | -7.208 | 02-04 to 22-08 | |
| Marine | Benbecula to South Uist | K | | 2 (2) | 57.391 | -7.269 | 02-04 to 22-08 | |
| Marine | South Uist to Eriskay | I | | 1 (2) | 57.092 | -7.270 | 02-04 to 22-08 | |
| Marine | Eriskay to Barra | I | | 9 (11) | 57.030 | -7.324 | 02-04 to 22-08 | |
| Marine | Vatersay to Sandray | I | | 2 (2) | 56.907 | -7.533 | 03-04 to 22-08 | |
| Marine | Sandray to Pabbay | I | | 2 (6) | 56.871 | -7.557 | 03-04 to 22-08 | |
| Marine | Pabbay to Mingulay | I | | 3 (4) | 56.840 | -7.601 | 03-04 to 22-08 | |
| Marine | Mingulay to Bernasay | I | | 1 (1) | 56.796 | -7.643 | 03-04 to 22-08 | |
| Marine | Lewis | L | | 8 (12) | 58.249 | -6.047 | 12-04 to 17-12 | |
| Marine | Sutherland | M | | 17 (18) | 58.506 | -5.248 | 13-04 to 30-08 | |
| Marine | Skye to Uist | J | | 58 (69) | 57.269 | -6.863 | 01-04 to 26-08 | |
| Marine | Islay to Jura | G | | 1 (1) | 56.141 | -5.629 | 31-03 to 29-08 | |
| Marine | Jura to mainland Scotland | G | | 6 (10) | 55.883 | -6.108 | 31-03 to 29-08 | |
| Marine | Skye to mainland Scotland | J | | 1 (2) | 57.224 | -5.654 | 31-03 to 29-08 | |
| Marine | Irish Sea | E | | 103 (112) | 55.494 | -6.886 | 27-02 to 13-08 | |
| Marine | Irish Sea | A | | 20 (22) | 55.931 | -5.477 | 19-03 to 03-08 | |
| Marine | North Atlantic Ocean (south of Hebridean Islands) | N | | 1 (1) | 56.604 | -7.855 | - | |
| Marine | North Atlantic Ocean (west of Hebridean Islands) | O | | 1 (1) | 57.098 | -8.969 | - | |
| Marine | North Atlantic (west of Hebridean Islands) | P | | 1 (1) | 58.0918 | -8.913 | - | |
| Marine | North Atlantic (Continental Shelf – submersible glider) | Q | | 1 (1) | 58.584 | -8.614 | 16-04 to 12-06 | |

Table S3. The total number of Atlantic salmon post-smolts detected at key monitoring points/ lines in this study. The statistics calculated in this table were calculated for each section of the migratory journey, which included the number of post-smolts detected at the start and end arrays and the date range post-smolts were detected on the end array for each section. The total duration (days) and Rate of Movement (RoM) between monitoring lines (body length per second (L_F_ s^-1^) / kilometres per day (km.day^-1^)) were also calculated. There were some instances where not all smolts detected on the end array were also detected on the start array, therefore, in these cases ROM was calculated using a proportion of the smolts detected. Note, Est – Estuary and CE – Coastal Embayment. This table expands on the data presented in a sister paper (Lilly et al. 2023).

| **River** | **Type** | **Start array** | **End array** | **Distance (km)** | **No. start array** | **No. end array (%)** | **Date range for detections at end array** | **No. used to calculate ROM** | **Mean RoM (L_F_ s^-1^) ± SD (range)** | **Mean RoM (km.day^-1^) ± SD (range)** | **Mean passage time (days) ± SD (range)** |
| --- | --- | --- | --- | --- | --- | --- | --- | --- | --- | --- | --- |
| Endrick | Est | Leven | C | 46.70 | 50 | 38 (76.00) | 28-04 to 31-05 | 38 | 0.81 ± 0.44  (0.17 - 2.14) | 10.14 ± 5.62  (2.12 - 27.61) | 4.75 ± 1.25  (3.30 - 16.21) |
|  | Mar | C | A | 86.80 | 38 | 4 (10.23) | 13-05 to 25-05 | 3 | 0.59 ± 0.24  (0.33 - 0.82) | 7.18 ± 2.62  (4.3 - 9.45) | 13.50 ± 9.19  (5.86 - 20.18) |
|  | Mar | C | E | 168.00 | 38 | 9 (23.68) | 12-05 to 18-06 | 9 | 0.77 ± 0.14  (0.55 - 0.96) | 9.69 ± 1.71  (7.11 - 11.95) | 17.86 ± 14.05  (3.37 - 23.63) |
| Gryffe | Est | Gryffe | C | 58.40 | 93 | 80 (86.02) | 22-04 to 22-05 | 80 | 1.06 ± 0.43  (0.3 - 2.22) | 13.65 ± 5.51  (3.64 - 27.56) | 5.18 ± 2.12  (2.66 - 16.05) |
|  | Mar | C | D | 27.90 | 80 | 4 (5.00) | 28-04 to 30-05 | 4 | 0.34 ± 0.17  (0.16 - 0.57) | 4.32 ± 2.37  (2.05 - 7.64) | 8 ± 3.65  (4.14 - 13.63) |
|  | Mar | C | A | 86.80 | 80 | 6 (7.50) | 06-05 to 04-06 | 6 | 0.46 ± 0.23  (0.18 - 0.82) | 6.05 ± 2.85  (2.38 - 10.33) | 17.80 ± 8.40  (10.02 - 36.48) |
|  | Mar | C | E | 168.00 | 80 | 27 (33.75) | 04-05 to 06-06 | 25 | 1.18 ± 0.47  (0.33 - 2.38) | 14.87 ± 5.59  (4.41 - 28.84) | 13.15 ± 5.83  (6.32 - 38.09) |
|  | Mar | A | E | 117 | 6 | 2 (33.33) | 14-05 to 06-06 | 2 | 0.37 ± 0.35 (0.12 – 0.62) | 2.24 ± 2.30 (0.79 – 4.05) | 17.80 ± 8.40 (10.02 -36.48) |
|  | Mar | C | B | 111.00 | 80 | 1 (1.25) | 15-05 | 1 | 0.4 | 6.77 | 16.39 |
|  | Mar | C | Q | 548 | 80 | 1 | 23-05 | 1 | 1.5 | 22.52 | 24.33 |
| Nith and Crawick | Mar | Nith | A | 170.00 | 90 | 20 (22.22) | 11-05 to 01-06 | 17 | 1.25 ± 0.44 (0.44 – 2.01) | 15.84 ± 5.49 (6.17 – 25.34) | 12.54 ± 5.94 (6.71 – 27.55) |
|  |  | A | E | 108.00 | 20 | 6 (30.00) | 19-05 to 06-06 | 3 | 2.69 ± 1.19 (1.34 – 3.62) | 36.70 ± 17.09 (18.89 – 51.29) | 3.59 ± 2.13 (2.11 – 6.04) |
|  |  | Nith | E | 278.00 | 90 | 6 (6.67) | 19-05 to 06-06 | 6 | 1.05 ± 0.49 (0.66 – 1.90) | 14.27 ± 7.03 (8.94 – 26.86) | 22.81 ± 8.45 (10.35 – 31.10) |
| Bladnoch | Mar | Bladnoch | A | 123.00 | 53 | 12 (22.22) | 13-05 to 06-06 | 9 | 1.05 ± 0.33  (0.77 - 1.66) | 12.85 ± 4.17 (9.71 - 20.69) | 10.28 ± 2.50  (5.95 - 12.66) |
|  |  | A | E | 108.00 | 12 | 5 (41.67) | 16-05 to 07-06 | 2 | 3.17 ± 0.6  (2.75 - 3.59) | 38.99 ± 2.77 (37.03 - 40.95) | 2.78 ± 0.2  (2.64 - 2.92) |
|  |  | Bladnoch | E | 238.00 | 53 | 5 (9.43) | 16-05 to 07-06 | 5 | 1.18 ± 0.38  (0.73 - 1.63) | 14.87 ± 4.17 (9.96 - 18.75) | 20.06 ± 6.14  (14.83 - 27.92) |
| Etive | Mar | Loch Etive | G | 15.30 | 62 | 22 (35.48) | 29-04 to 22-05 | 21 | 1.22 ± 0.70 (0.42 – 3.07) | 14.56 ± 8.25 (4.83 – 34.98) | 1.46 ± 0.87 (0.44 – 3.17) |
|  | Mar | Loch Etive | H | 24.20 | 62 | 6 (9.70) | 30-04 to 23-05 | 5 | 0.99 ± 0.31 (0.56 – 1.43) | 11.93 ± 4.66 (6.43 – 19.29) | 2.29 ± 0.92 (1.25 – 3.76) |
|  | Mar | Loch Etive | J | 172.00 | 62 | 1 (1.61) | 27-05 | 0 | - | - | 14.16 |
|  | Mar | Loch Etive | N | 159.00 | 62 | 1 (1.61) | 25-05 | 0 | - | - | 11.53 |
| Orchy | Mar | Loch Etive | G | 15.30 | 90 | 24 (26.67) | 23-04 to 24-05 | 23 | 1.39 ± 0.63 (0.38 – 2.57) | 16.38 ± 7.47 (4.29 – 30.43) | 1.27 ± 0.87 (0.50 – 3.56) |
|  | Mar | Loch Etive | H | 24.20 | 90 | 17 (18.89) | 28-04 to 23-05 | 15 | 1.32 ± 0.53 (0.56 – 0.23) | 16.72 ± 7.15 (6.99 – 32.60) | 1.75 ± 0.83 (0.74 – 3.46) |
|  | Mar | Loch Etive | E | 139.00 | 90 | 1 (1.11) | 15-05 | 1 | 1.30 | 16.26 | 8.55 |
|  | Mar | Loch Etive | I | 142.00 | 90 | 2 (2.22) | 11-05 to 13-05 | 2 | 1 | 11.62 | 11.40 |
|  | Mar | Loch Etive | J | 137.00 | 90 | 1 (1.11) | 07-05 | 1 | 0.73 | 9.61 | 14.16 |
|  | Mar | Loch Etive | Q | 362 | 90 | 1 | 29-05 | 1 | 1.48 | 17.87 | 20.25 |
| Loy and Lundy | Mar | Loch Linnhe | G | 53.00 | 101 | 33 (32.67) | 02-05 to 21-07 | 27 | 1.75 ± 0.63 (0.87 – 3.36) | 20.70 ± 7.20 (10.12 – 38.05) | 2.87 ± 1.0 (1.39 – 5.23) |
|  |  | Loch Linnhe | H | 52.00 | 101 | 48 (47.52) | 21-04 to 20-07 | 42 | 2.14 ± 0.79 (0.85 – 4.18) | 25.16 ± 9.26 (9.92 – 46.96) | 2.41 ± 1.04 (1.11 – 5.24) |
|  |  | G | J | 234.00 | 101 | 1 (0.99) | 14-05 | 1 | 1.19 | 14.02 | 16.69 |
|  |  | H | J | 172.00 | 101 | 1 (0.99) | 02-05 to 08-05 | 1 | 1.68 | 19.56 | 8.79 |
| Badnabay | Mar | Loch Laxford | M | 11.70 | 4 | 2 (50.00) | 02-05 to 11-05 | 1 | 1.88 | 22.78 | 0.51 |
| Laxford | Mar | Loch Laxford | M | 11.70 | 62 | 27 (43.55) | 01-05 to 17-05 | 22 | 2.52 ± 0.94 (1.12 – 4.44) | 31.07 ± 12.08 (13.16 – 53.38) | 0.44 ± 0.19 (0.22 – 0.89) |
| Laxay | Mar | Loch Eireasort | L | 22.80 | 64 | 12 (18.75) | 22-04 to 17-05 | 6 | 1.40 ± 0.41 (0.63 – 1.73) | 16.93 ± 5.32 (7.20 – 21.19) | 1.55 ± 0.81 (1.08 – 3.16) |
|  | Mar | Loch Eireasort | M | 74.30 | 64 | 13 (20.31) | 02-05 to 17-05 | 13 | 1.07 ± 0.42 (0.50 – 2.09) | 13.45 ± 5.1  (6.75 – 24.93) | 6.30 ± 2.41 (2.98 – 11.01) |
| Roe | Est | Roe | Lough Foyle | 14.80 | 9 | 6 (66.67) | 05-05 to 13-05 | 3 | 0.4 ± 0.25  (0.12 - 0.6) | 5.38 ± 3.3  (1.62 - 7.78) | 4.41 ± 1.9  (4.1 -9.15) |
|  | Mar | Lough Foyle | E | 37.80 | 6 | 5 (83.33) | 06-05 to 13-05 | 5 | 3.08 ± 0.9  (2.21 - 4.05) | 39.94 ± 12.04 (27.69 - 52.93) | 1.02 ± 0.71  (0.3 - 1.37) |
| Faughan | Est | Faughan | Lough Foyle | 27.00 | 38 | 19 (50.00) | 12-05 to 02-06 | 19 | 1.13 ± 0.4  (0.69 - 1.88) | 13.75 ± 4.76 (8.68 - 23.11) | 2.16 ± 1.17  (0.66 - 3.11) |
|  | Mar | Lough Foyle | E | 37.8 | 19 | 18 (94.73) | 13-05 to 01-06 | 10 | 2.39 ± 1.19  (0.33 - 4.45) | 29.56 ±15.30 (3.87 - 55.78) | 2.24 ± 0.68  (2.74 - 9.76) |
| Bush | CE | Bush | Runkerry Bay | 1.81 | 73 | 64 (87.67) | 18-04 to 09-05 | 64 | 2.43 ± 2.14  (0.05-8.35) | 35.22 ± 30.75 (0.7 - 119.1) | 0.19 ± 0.36  (0.02 - 2.58) |
|  | Mar | Runkerry Bay | E | 45.3 | 64 | 39 (60.94) | 07-05 to 29-04 | 37 | 2.02 ± 0.82  (0.45 - 4.17) | 29.44 ± 12.23 (7.51 - 59.81) | 1.9 ± 1.07  (0.76 - 6.04) |
|  | Mar | Runkerry Bay | G | 124 | 64 | 1 (1.56) | 28-04 | 1 | 1.03 | 16.23 | 7.64 |
| Glendun | Mar | Glendun | E | 83.40 | 21 | 5 (23.81) | 02-05 to 15-05 | 5 | 1.34 ± 0.24 (1.08 - 1.74) | 16.39 ± 3.25 (12.09 - 21.04) | 2.97 ± 0.61 (2.24 - 3.90) |
|  | Mar | Glendun | I | 233.00 | 21 | 1 (4.76) | 05-06 | 0 | - | - | - |
|  | Mar | Glendun | P | 391.0 | 21 | 1 | 19-05 | 1 | 1.93 | 25.87 | 15.11 |
|  | Mar | Glendun | G | 150.0 | 21 | 1 | 26-05 | 1 | 0.51 | 6.57 | 22.84 |
| Bann and Agivey | Mar | Bann | E | 45.60 | 34 | 21 (61.76) | 05-05 to 26-05 | 17 | 2.79 ± 0.93  (1.47 - 4.67) | 38.56 ± 12.86 (20.29 - 61.64) | 1.31 ± 0.44  (0.74 to 2.25) |
|  | Mar | Bann | Q | 402.0 | 34 | 1 | 31-05 | 1 | 1.7 | 22.10 | 18.19 |
| Carey | Mar | Carey | E | 60.40 | 7 | 3 (60.00) | 05-05 to 07-05 | 2 | 0.99 ± 0.28  (0.8 - 1.19) | 14.18 ± 4.89 (10.73 - 17.64) | 4.53 ± 1.56  (3.42 - 5.63) |
| Derwent | Mar | Derwent | A | 139.00 | 41 | 15 (36.58) | 11-05 to 06-06 | 3 | 0.98 ± 0.53  (0.6 - 1.58) | 11.56 ± 6.01 (6.86 - 18.33) | 14.17 ± 6.35  (7.58 - 20.25) |
|  | Mar | Derwent | C | 235.00 | 41 | 1 (2.44) | 25-06 | 1 | 0.72 | 9.1 | 25.83 |
|  | Mar | Derwent | D | 218.00 | 41 | 3 (7.32) | 06-06 to 13-07 | 2 | 0.63 ± 0.11  (0.55 - 0.7) | 7.76 ± 1.6  (6.63 - 8.89) | 28.71 ± 5.91  (24.53 - 32.89) |
|  | Mar | Derwent | E | 256.00 | 41 | 11 (26.83) | 13-05 to 20-06 | 7 | 1.18 ± 0.61  (0.46 - 2.18) | 14.03 ± 7.32 (5.52 - 25.28) | 23.04 ± 12.14 (10.13 - 46.53) |
|  | Mar | Derwent | P | 564.00 | 41 | 1 (2.44) | 08-06 | 0 | - | - | - |
| Burrishoole (Wild) | CE | Burrishoole estuary | Clew Bay 1 | 4.89 | 17 | 15 (88.24) | 07-05 to 15-05 | 14 | 1.94 ± 1.25 (0.23 - 3.95) | 25.31 ± 16.90 (2.87 - 54.17) | 0.37 ± 0.42 (0.09 - 1.71) |
| (Ranched) | CE | Burrishoole estuary | Clew Bay 1 | 4.89 | 51 | 37 (72.55) | 06-05 to 05-06 | 35 | 1.40 ± 1.57 (0.01 - 4.22) | 23.72 ± 26.48 (0.16 - 72.59) | 3.47 ± 6.09 (0.07 - 30.27) |
| (Wild) | CE | Burrishoole estuary | Clew Bay2 | 25.70 | 17 | 7 (41.18) | 12-05 to 22-05 | 7 | 1.77 ± 1.30 (0.23 - 3.29) | 23.08 ± 16.81 (2.83 - 43.18) | 2.72 ± 3.17 (0.60 - 9.07) |
| (Ranched) | CE | Burrishoole estuary | Clew Bay 2 | 25.70 | 51 | 15 (29.41) | 09-05 to 15-05 | 15 | 1.50 ± 1.01 (0.37 - 2.91) | 25.44 ± 17.39 (6.61 - 53.71) | 1.64 ± 1.09 (0.48 - 3.89) |
| (Ranched) | Mar | Clew Bay 2 | N | 364 | 15 | 2 (13.33) | 24-05 | 0 | - | - | - |
| (Ranched) | Mar | Clew Bay 2 | O | 386 | 15 | 1 (6.67) | 06-05 | 1 | 0.75 | 65 | 5.92 |
| (Ranched) | Mar | Clew Bay 2 | Q | 541 | 15 | 1 (6.67) | 04-06 | 1 | 0.24 | 20.74 | 26.09 |
| Torridon | Mar | Loch Torridon | M | 118 | 3 | 2 (66.67) | 06-05 to 11-05 | 1 | 1.50 ± 0.80 (0.94 - 2.06) | 18.13 ± 10.41 (10.77 - 25.49) | 7.79 ± 4.48 (4.63 - 10.96) |
| Balgy | Mar | Loch Torridon | L | 81.50 | 5 | 1 (20) | 01-05 | 1 | 1.46 | 19.39 | 4.20 |
|  | Mar | Loch Torridon | M | 118 | 5 | 4 (80) | 10-05 to 13-05 | 4 | 0.12 ± 0.03 (0.09 - 0.15) | 11.64 ± 3.23 (0.09 - 15.98) | 10.69 ± 2.68 (7.39 - 12.99) |
| Shimna | Mar | Shimna | A | 101 | 3 | 1 (33.33) | 31-05 | 1 | 0.33 | 4.54 | 22.27 |
| Boyne | Mar | Boyne | A | 152.89 | 63 | 12 (19.05) | 15-05 to 03-06 | 7 | 0.64 ± 0.35  (0.29 – 1.37) | 8.87 ± 4.84  (4.55 – 19.36) | 20.48 ± 7.9  (7.89- 34.03) |
| Boyne | Mar | A | D | 82 | 12 | 1 (8.33) | 28-05 | 1 | 1.05 | 15.93 | 5.14 |
| Boyne | Mar | Boyne | E | 265 | 63 | 5 (7.94) | 20-05 to 10-06 | 5 | 0.76 ± 0.16  (0.62 – 1.01) | 10.52 ± 2.25  (8.70 – 13.92) | 26.03 ± 5.00  (19.04 – 30.47) |
| Boyne | Mar | A | E | 110 | 12 | 1 (8.33) | 06-06 | 1 | 2.91 | 45.94 | 2.39 |
